# Domain duplication, divergence, and loss events in vertebrate Msx paralogs reveal phylogenomically informed disease markers

**DOI:** 10.1186/1471-2148-9-18

**Published:** 2009-01-20

**Authors:** John R Finnerty, Maureen E Mazza, Peter A Jezewski

**Affiliations:** 1 Department of Biology, Boston University, 5 Cummington Street, Boston, MA 02215, USA; 2 Department of Oral Medicine, Infection and Immunity, Harvard School of Dental Medicine, Boston, MA, USA; 3 Department of Cytokine Biology, The Forsyth Institute, 140 The Fenway, #411, Boston, MA 02115, USA

## Abstract

**Background:**

Msx originated early in animal evolution and is implicated in human genetic disorders. To reconstruct the functional evolution of Msx and inform the study of human mutations, we analyzed the phylogeny and synteny of 46 metazoan Msx proteins and tracked the duplication, diversification and loss of conserved motifs.

**Results:**

Vertebrate Msx sequences sort into distinct Msx1, Msx2 and Msx3 clades. The sister-group relationship between *MSX1 *and *MSX2 *reflects their derivation from the 4p/5q chromosomal paralogon, a derivative of the original "MetaHox" cluster. We demonstrate physical linkage between Msx and other MetaHox genes (*Hmx*, *NK1*, *Emx*) in a cnidarian. Seven conserved domains, including two Groucho repression domains (N- and C-terminal), were present in the ancestral Msx. In cnidarians, the Groucho domains are highly similar. In vertebrate Msx1, the N-terminal Groucho domain is conserved, while the C-terminal domain diverged substantially, implying a novel function. In vertebrate Msx2 and Msx3, the C-terminal domain was lost. MSX1 mutations associated with ectodermal dysplasia or orofacial clefting disorders map to conserved domains in a non-random fashion.

**Conclusion:**

Msx originated from a MetaHox ancestor that also gave rise to Tlx, Demox, NK, and possibly EHGbox, Hox and ParaHox genes. Duplication, divergence or loss of domains played a central role in the functional evolution of Msx. Duplicated domains allow pleiotropically expressed proteins to evolve new functions without disrupting existing interaction networks. Human missense sequence variants reside within evolutionarily conserved domains, likely disrupting protein function. This phylogenomic evaluation of candidate disease markers will inform clinical and functional studies.

## Background

The Msx gene family is one of the oldest animal-specific homeodomain transcription factors. Msx genes have been identified in basal, *i.e*. diploblastic, animals such as sea anemones [1,2], corals [3], hydras [4,5] jellyfishes [6], and sponges [7,8] They have also been described from eleven different phyla of triploblastic animals [9].

Since its origin at or near the base of the Metazoa, Msx appears to have evolved in a relatively conservative fashion. The locus has not undergone the rampant gene duplication seen in a number of other Antennapedia-class homeodomain genes, although vertebrates are known to possess two (human), three (mouse), or five (zebrafish) Msx paralogs. Furthermore, at least one portion of the protein has been extremely highly conserved – *e.g*., only two of the 60 positions in the homeodomain differ between *Nematostella *(a sea anemone) and *Branchiostoma *(a chordate), two taxa that diverged over 600 million years ago. Concordant with the conservative molecular evolution of this developmental regulatory protein, Msx appears to have retained an ancient role in neuro-ectodermal patterning and differentiation in vertebrates, arthropods, and perhaps cnidarians [4,10,11]. Msx proteins are also consistently expressed at sites of epithelial-mesenchymal interactions [12-14]. Msx, NK, and Tlx homeobox genes share common expression patterns during early dorso-ventral neurectodermal and mesodermal development as well as during anterior-posterior segmentation events, in both flies (Ecdysozoa) and the slowly evolving nereid annelids (Lophotrochozoa) [15], that resemble dorsal-ventral expression patterns found during development in vertebrates (Deuterostomia) [16]. This is especially notable since Msx genes are found clustered with NK and Tlx homeobox genes in large MetaHox clusters or paralogons [17-19].

Msx genes have also assumed diverse developmental roles in vertebrates and arthropods, and they are known to have played key roles in the evolution of novel ontogenies and novel morphologies. For example, altered expression of Msx genes has been implicated in the evolution of direct development in sea urchins [20] and caudal fin elaboration of male sword-tailed fishes [21]. The expansion of the Msx family in vertebrates via gene duplication has been accompanied by divergent expression patterns between Msx paralogs [22], and perhaps by an overall expansion of Msx-mediated developmental processes.

Msx1 and Msx2 exhibit both redundant and complementary spatiotemporal expression patterns and protein functions during vertebrate development [23-31]. In vertebrates, Msx1 protein is pleiotropically expressed in a range of craniofacial structures including neural crest, branchial arches and sensory placodes. Msx1 is also expressed during fin/limb bud outgrowth and during early gastrulation, as well as at sites of ectodermal-mesenchymal interactions. Mouse Msx1 and Msx2 are both expressed in migrating cranial neural crest cells.

The two Msx genes in humans, *MSX1 *and *MSX2*, are both important in human genetic disorders. Mutations in these genes have been identified in individuals exhibiting both syndromic/Mendelian and nonsyndromic/complex genetic disorders. Human MSX1 coding mutations have been identified in patients with either orofacial clefting (OFC) [32-34], ectodermal dysplasias (ED), (such as tooth agenesis and nail malformation) [35-37], or both phenotypes [38]. By contrast, human MSX2 mutations are predominantly associated with cranial malformations [39-41], although murine studies suggest a role for MSX2 in bone and ectodermal organ formation [42].

In order to understand how the developmental roles of the Msx genes were altered by gene duplication in vertebrates, a better understanding of Msx gene family evolution in vertebrates is needed. At this time, our understanding is quite limited. For example, the precise relationship among the mammalian and teleost paralogs has not been convincingly established [22,43].

In analyzing putative human Msx mutations, the greatest challenge may lie in distinguishing neutral genetic variation from mutations that are likely to have significant clinical consequences in multifactorial disease cases [44]. Given the complex and sometimes overlapping spatiotemporal expression patterns of different Msx paralogs in vertebrates, unraveling the phenotypic consequences of particular Msx mutations is made even more difficult. In recent years, with the proliferation of DNA sequence data, it has become possible to consider the degree of evolutionary conservation when predicting the phenotypic consequences of sequence variation. For example, Kashuk and co-workers found those missense mutations that mapped to evolutionarily invariant positions in an alignment of RET proteins were more likely to be associated with the most severe clinical outcomes [45].

In an effort to identify MSX1 mutations that are most likely to have important phenotypic consequences, we undertook an evolutionary analysis of diverse vertebrate Msx genes. A recent analysis of Msx genes from 13 different animal phyla [9] identified five conserved coding domains. These included two Groucho-binding domains, a conserved motif upstream of the conserved intron, the homeodomain and its C-terminal flanking region, with all but the duplicate Groucho domain having been previously noted [46-48]. Using a different approach and a different selection of taxa, we have determined that seven conserved coding domains were present in the common ancestor of all eumetazoan Msx genes, including those reported by Takahashi and co-workers. These included a set of conserved residues located both upstream and downstream of the homeodomain, a Pbx binding motif and a PIAS-binding domain located at the carboxy terminus. We also provide evidence for the derivation of these coding domains from an ancestral MetaHox cluster gene.

While all seven domains are widely conserved in metazoan Msx proteins, the duplicate vertebrate Msx proteins differ strikingly with respect to their Groucho repression domains. Relative to Msx1, both Msx2 and Msx3 diverged slightly more rapidly in the N-terminal Groucho repression domain. However, the C-terminal Groucho domain appears to have been substantially modified and was likely independently lost in both Msx2 and Msx3, while it has evolved only slightly from the inferred ancestral sequence in Msx1. The functional evolution of these domains is likely critical to understanding the nature of Msx mutations, as the two main phenotypic categories of MSX1 mutants – (1) ectodermal dysplasias and (2) oral/facial clefting disorders – are not randomly distributed across the length of the protein. The evolutionary analysis also permits us to identify those human sequence variants that are most radical when evaluated against the background of Msx evolutionary history. Since such mutations run counter to long-standing stabilizing selection acting upon Msx, they are likely to have deleterious phenotypic consequences.

In addition to its medical relevance, Msx evolution has wider implications for the origins of biological novelty. Cis-regulatory evolution is thought to be the most common driver of morphological innovation, with protein evolution being a less common cause due to stronger stabilizing selection acting on protein sequences [49]. In a pleiotropically expressed regulatory protein like Msx, any change in the coding sequence has the potential to impact regulatory interactions in multiple temporal and spatial contexts, so any deleterious effect will be magnified. In such proteins, functionally significant residues will be under very strong stabilizing selection [50]. The constraints acting on protein sequence may however be relaxed by genome and gene duplication events [51-54]. We discuss how the evolution of Msx incorporated an additional layer of complexity because early in its history, Msx underwent a domain duplication, and similar to duplicate genes or duplicate cis-regulatory modules, duplicate domains encode the possibility of functional redundancy. The differences between Msx1 and Msx2 point to a duplication and subsequent functional divergence of the Groucho repression domains as being a key feature in their evolution that help define the mutation phenotype patterns.

## Results

### Msx domains and motifs

Logan et al., 1992 compared Engrailed paralogs from diverse vertebrate species and identified five conserved coding domains [55]. They named these domains the EH1-5 for Engrailed Homology domains, 1–5. Subsequent work has identified similar sequence motifs and functional domains within many other proteins, most notably transcription factors [46,56-60], that included the Msx family [46,48].

**Figure 1:**
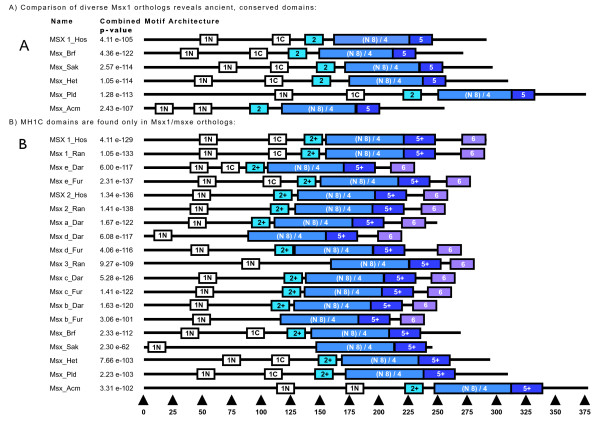
**MEME Domain Identification in the Msx Family**. A) Conserved domains identified by MEME in a comparison of human MSX1 (Msx1_Hos) and the Msx proteins of cephalochordate, hemichordate, sea urchin, polychaete, and coral. The domains are named consecutively from the N-terminal to the C-terminal ends of the protein as Msx Homology (MH) domains 1–5. B) Conserved domains identified by MEME in a comparison of vertebrate Msx paralogs plus single Msx proteins from cephalochordate, tunicate, sea urchin, polychaete, and coral. The MH2 and MH5 domains include more amino acids (see Fig. 2) due to the increased sequence similarity within the vertebrate clade. These slightly larger domains are indicated by a "+". Taxon/gene abbreviations are listed in the methods.

**Figure 2:**
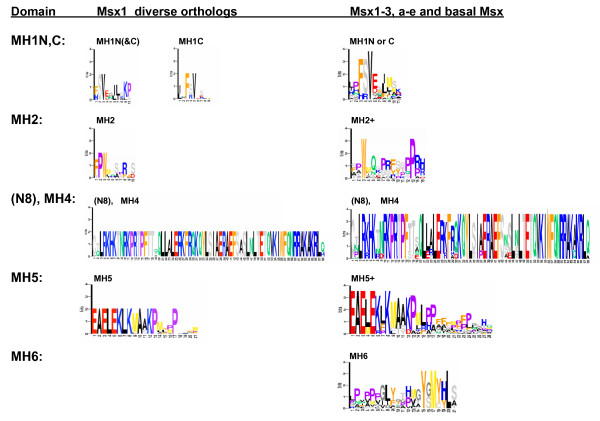
**LOGO Position Specific Scoring Matrices (PSSMs) identified by MEME**. The first column shows the LOGO PSSMs identified for Msx1 orthologs from the MEME result shown in Fig. 1A; the second column shows the LOGO PSSMs identified for vertebrate Msx1, 2, 3 and Msx sequences from basal taxa from the MEME result shown in Fig. 1B. Note that the LOGO motif labeled "MH1N(&C)" refers to all the N-terminal MH1 domains in Figure 1A plus the C-terminal domain in the coral, *Acropora*. The second column LOGO labeled "MHIN or C" was a consensus motif found among the predominantly vertebrate taxa. The slightly longer MH2 and MH5 domains in this second column are indicated with a "+". Also note that the LOGOs on the third row illustrate the contiguous N8 amino acids plus the MH4 homeodomain. This N8 is considered as a portion of the MH3 linker that spans the MH2 and MH4 domains, as described in the text.

To look for common coding domains relevant to the evolution of the Msx family, comparisons were deliberately made between Msx sequences from taxa more ancient than that utilized in the engrailed comparisons, since current evidence suggests that the Msx family is more ancient than the engrailed family [61]. First, to look for ancient sequence homologies relevant to deuterostome taxa, human MSX1 was compared with Msx proteins from a cephalochordate, a urochordate, an echinoderm, and two non-deuterostomes that served as outgroups, a nereid annelid [62] and a cnidarian. Five conserved domain-types were identified in this initial analysis that closely resemble those from the Engrailed family. These domains may actually be a plesiomorphic character relative to the origin of the Engrailed family. Thus these domains were designated as Msx Homology domains 1 to 5, or MH1-5 (Fig. 1A, 2), in parallel to the nomenclature for the Engrailed domains. All five of these Msx proteins were found to harbor two MH1 domains, labeled as MH1N and MH1C, (named for their respective positions within the protein nearer the amino (N) or carboxy (C) terminus of the protein).

Secondly, to probe for the vertebrate lineage-specific homologies, mammalian Msx1, Msx2, and Msx3 protein sequences were compared with Msx proteins sequences from three bony fishes, a cephalochordate, two urochordates, an echinoderm, and again, the nereid annelid. This latter sequence was included here to represent a slowly evolving, non-deuterostome outgroup [62,63]. A novel result from this analysis was the identification of an additional conserved domain at the C-terminus of the vertebrate Msx proteins designated MH6 (Figs. 1B, 2). While MH6 was not identified directly in the initial analysis of the non-vertebrate taxa, multi-sequence alignments reveal that many core amino acids within the MH6 domain are also conserved in these animals (Additional file 1 page 8).

The most striking feature discovered with the vertebrate sequence set was that only the vertebrate Msx1 or Msxe orthologs encode both the MH1N and MH1C domains. According to the MEME analysis, the Msx2, Msxd, Msxa, Msx3, Msxb and Msxc proteins lack the MH1C domain (See Fig. 1B). As both MH1N and MH1C domains are present in non-vertebrate taxa, including basal metazoans such as the coral and the sea anemone, these data suggest that retention of both domains in Msx1 or Msxe orthologs is the primitive condition, making them more similar in sequence and perhaps function, to the ancestral Msx protein and the single Msx homologs found in extant basal animals.

Putative functions can be ascribed to each of these deeply conserved domains based upon their strong sequence resemblance to motifs in other closely related homeodomain proteins that have already undergone functional analysis (Fig. 2) [23,47,56,64-72]. Such comparisons suggest that the MH1N and MH1C domains are Groucho repression domains. The engrailed EH2 domain includes a motif that resembles the "hexapeptide," a motif first identified in Hox proteins and that is known to augment DNA binding specificity by binding to Pbx family proteins as a cofactor [56]. However, relative to the "hexapeptide" of Hox genes, the engrailed Pbx-binding motif has an extra crucial tryptophan residue. Interestingly, the MH2 domains in the Msx proteins of basal metazoans and non-vertebrate deuterostomes most often have double tryptophans, while the vertebrate Msx proteins have an MH2 that resembles the hexapeptide motifs of anterior Hox genes in having only a single tryptophan [73].

The MH3 domain defined here corresponds to the EH3 domain of engrailed proteins. Basically, MH3 is the linker between the MH2 domain and the homeodomain. This linker includes the eight highly conserved amino acids immediately upstream of the homeodomain (labeled "N8" in Figs. 1, 2). Our rationale for defining this whole span as MH3 is based on how the conservation observed within our multisequence alignments (Additional File 1 page 4–5) corresponds to both functional data from engrailed's EH3 domain [56], as well as additional conserved phosphorylation motifs [74], previously identified in Hox proteins [75].

The MH4 motif corresponds to the homeodomain, which is known to be involved in DNA binding and protein-protein (homo- or hetero-) dimerization. MH5, which is contiguous with the carboxy-terminus of the homeodomain, has been shown to be involved in transcriptional repression. Finally, MH6 appears to be the PIAS protein-binding domain.

To investigate the generality and antiquity of the pattern of conserved Msx homology domains identified by MEME, we used a motif-based Hidden Markov Model of these domains (generated using MetaMEME) to search a manually assembled sequence collection as well as online databases. The manually assembled sequence collection comprised two chordate Msx proteins, two cnidarian Msx proteins, and two poriferan Msx proteins in addition to full-length NK and Tlx homeobox proteins, including all of the published full-length MetaHox protein sequences from sponges. Representative MetaMEME results are shown in Additional File 2. As expected, the full set of Msx Homology (MH) domains was re-identified within the chordate and cnidarian Msx sequences, evidenced by the high match scores (165–399) and by the presence of those most highly conserved core amino acid residues within the LOGO position specific scoring matrices illustrated in Fig. 2. By contrast, the highest match scores to non-Msx protein sequences (65–128) were to NK1/NK2-3-4, Tlx, and BarH/Bsh proteins. The match scores and conserved residues found within the Msx sequence of *Amphimedon queenslandica *(a sponge) reveal strong matches to the MH3/4 domains and much weaker matches to the MH1 and MH2 domains, that lack canonical cores amino acids.

It is also notable that there is a strong match, although not scored by this initial MetaMEME analysis, between the 12 amino acid "R1" sequence found downstream of several Demox homeodomain proteins (EETEMEMKSPKY) [59], and the first portion of the MH5 canonical sequence (EAELEKLKMAAKPMLPPGLFM) found in Msx proteins. It is the first thirteen amino acid residues of the MH5 domain that is the most conserved (Fig. 2). It is possible to use slightly different parameter settings within MEME that limit domain sizes. When this is done, a smaller canonical MH5 domain is obtained that displays just these most conserved residues. MetaMEME then identifies strong matches between the MH5 domain of vertebrate Msx proteins and the AmqMsx as well as the R1 domain of Tlx and Demox proteins (data not shown).

When we searched the much larger non-redundant database of proteins sequences for Msx Homology (MH) domains using MetaMEME, as expected, the highest match scores were again found for Msx sequences (248–403) with significance scores ranging from e-52 to e-85. When attempting to identify non-Msx proteins possessing the same conserved domains, we first screened the results to eliminate those sequences without homeodomains and those sequences with low-scoring domains occupying different relative positions. The sequences that met these criteria and scored the highest matches to the Msx homology domains were Emx, Hmx (an NK gene), Engrailed, Dlx, Gbx, Nk, and Tlx; the highest scores were in the range of 113 to 67, with significance values in the range of e-19 down to e-10. Within these non-Msx proteins, strong matches to particular domains were also identified, including MH1, MH2, MH3/4, and MH5, as above. Similar results were obtained using the MAST program, the motif based local alignment tool within the MEME suite of programs (data not shown).

### Multisequence alignment of Msx protein sequences

A full-length multisequence alignment of all the Msx orthologs and paralogs was facilitated by the identification of the conserved Msx Homology domains, as highly conserved amino acids within MH domains (MH1N and MH2) were used as homologous landmarks to keep the alignments in register (see Methods). Amino acid sequences either upstream, downstream or in between these domains were then aligned en bloc by the Clustal algorithm within MEGA 4.0 [76]. Thus the final full sequence alignment has input from both local and global alignment algorithms.

The full sequence alignments of all Msx orthologs and paralogs (Additional File 1 page 1–8) revealed that the coding region that contained the MH1C domain in Msx1 and Msxe orthologs appeared to be deleted or highly diverged in the Msx2/a/d and Msx 3/b/c paralog groups (Additional File 1 page 4). This corroborates the result from the MEME analysis (Fig. 1B), and suggests the MH1C domain was lost prior to the diversification of the Msx2/a/d and Msx 3/b/c genes, likely by some combination of deletion/subfunctionalization and neofunctionalization [52,77,78].

### Phylogenetic analysis

**Figure 3:**
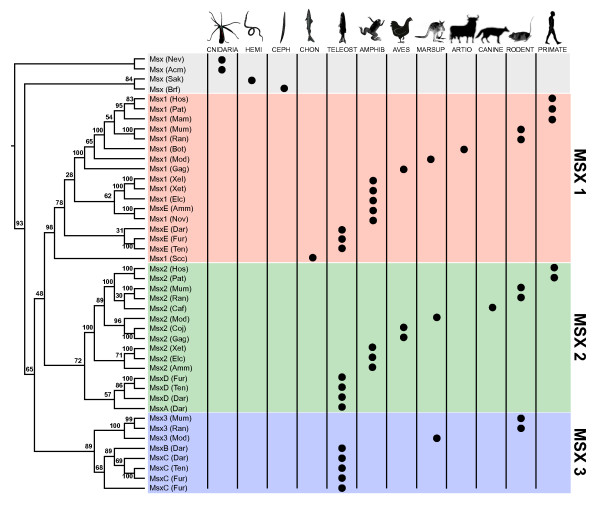
**Msx Phylogeny based upon the full Msx alignment**. Relationships among 44 metazoan Msx proteins were estimated by neighbor-joining (see methods). The tree is rooted using the two cnidarian sequences. Numbers at nodes indicate the percentage of replicates in which a given partition between taxa was observed in 1000 replicates of the bootstrap [106]. Circles indicate the major taxonomic group represented by each sequence (Hemi = Hemichordata, Ceph = Cephalochordata, Chon = Chondrichthyes, Amphib = Amphibia, Marsup = Marsupialia, Artio = Artiodactyla). Species abbreviations are provided in the methods.

A neighbor-joining analysis of 44 Msx proteins is presented in Fig. 3. The tree depicted is based upon the full alignment and is rooted using the two cnidarian Msx sequences. All of the vertebrate Msx sequences appear as a monophyletic group, and within this vertebrate Msx clade, we can recognize distinct Msx1, Msx2, and Msx3 lineages. From the distribution of placental, marsupial, avian, amphibian, teleost, and chondrichthyan sequences among these three clades, we can conclude that the Msx1, 2, and 3 lineages had diverged prior to the evolutionary split between bony fishes and cartilaginous fishes. Furthermore, it appears that the Msx1 and Msx2 lineages share a common ancestor to the exclusion of Msx3. If we map the presence of conserved Msx domains on this phylogeny, it appears that MH1C has been lost independently in both the Msx2 and Msx3 families of vertebrates.

We also performed a phylogenetic analysis after removing all characters that harbored alignment gaps. A phylogeny is presented in Additional File 3 based upon this gap-free alignment (Additional file 4). As in the full alignment, the gap-free alignment supports a sister-group relationship between an Msx1 clade and an Msx2 clade. However, the gap free analysis does not support the monophyly of a clade comprising tetrapod Msx3 genes and teleost MsxA/D genes. Rather, the teleost genes and the tetrapod genes emerge as two independent lineages at the base of the vertebrate Msx radiation

**Figure 4:**
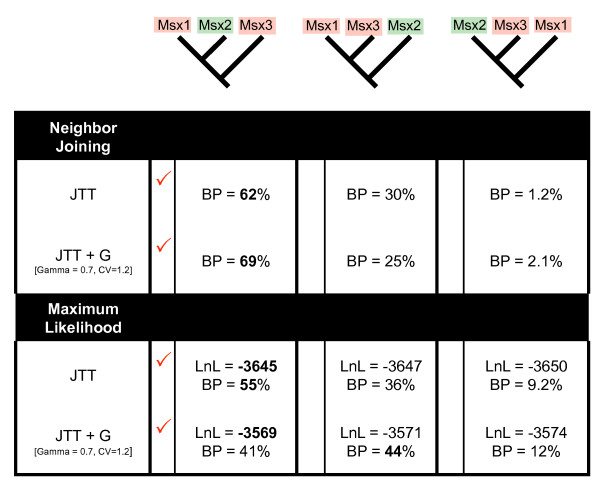
**Phylogenetic support for alternate relationships among Msx1, Msx2, and Msx3**. The three possible relationships among Msx1, Msx2, and Msx3 were directly compared using a dataset consisting of 11 taxa. Trees were generated using neighbor-joining and maximum-likelihood, with and without variation among sites (see methods). In all four instances, the favored topology grouped Msx1 with Msx2 to the exclusion of Msx3 (check marks).

To compare the relative support for the three possible relationships among Msx1, Msx2, and Msx3 proteins, we conducted a battery of phylogenetic analyses on a subset of the taxa (see Methods). Both neighbor-joining and maximum-likelihood analyses of this smaller dataset, performed both with and without rate variation among sites, support the grouping of Msx1 with Msx2 (Fig. 4). Given the extremely low bootstrap support for the grouping of Msx2 and Msx3, this possibility can be confidently ruled out. However, the bootstrap analyses reveal some support in the data for the grouping of Msx1 with Msx3, and in one instance (a maximum-likelihood analysis assuming rate variation among sites), the bootstrap support for this hypothesis actually exceeds the support for an Msx1-Msx2 clade. Importantly, the grouping of Msx1 with either Msx2 or Msx3 would imply that the MH1C motif has been independently lost in Msx2 and Msx3.

### Evolution of the Groucho-binding domains (MH1N and MH1C) in vertebrate Msx paralogs

**Figure 5:**
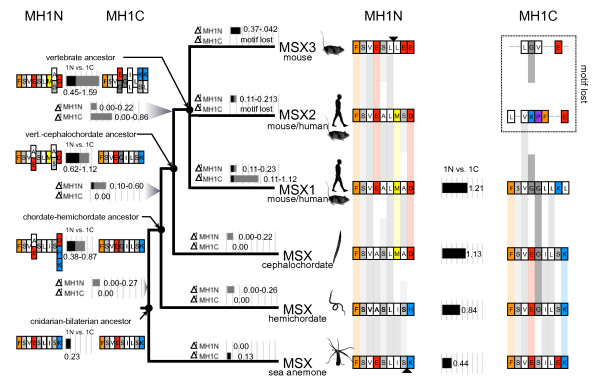
**Divergence and Loss of Msx Groucho-binding Domains (MH1N and MH1C)**. The ancestral sequences of MH1N and MH1C were inferred for four key ancestors (solid circles) based on sequences found in extant animals (right). Single alignment gaps were removed from mouse Msx3 and *Nematostella *Msx (triangles). Estimated evolutionary distances were calculated from each ancestor to its descendent(s) (ΔMH1N and ΔMH1C). Where MacClade inferred multiple possible ancestral states, the range of possible evolutionary distances is given. The evolutionary distance between MH1N and MH1C was calculated for each ancestor and each extant animal (1N vs. 1C). Distance calculations were not made to MH1C in Msx2 and Msx3 because no significant match to the MH1C motif was identified in these proteins.

The MEME analysis, the alignment, and the phylogenetic analysis indicate that the MH1 domain duplicated early in the evolution of the Msx family, prior to the divergence of Cnidaria and Bilateria, and that the MH1C domain was subsequently lost or underwent extensive sequence divergence in the Msx2/a/d and Msx 3/b/c lineages. In an attempt to reconstruct the divergence of these duplicate Groucho-binding domains over the course of vertebrate evolutionary history, we used parsimony to infer the ancestral sequences of MH1N and MH1C in four key ancestors: (1) the vertebrate ancestor, (2) the vertebrate-cephalochordate ancestor, (3) the chordate-hemichordate ancestor, and (4) the cnidarian-bilaterian ancestor. We then calculated the evolutionary distance from these hypothetical ancestral sequences to the modern day Msx paralogs of rodents and primates and the single Msx sequences in the cephalochordate, *Branchiostoma floridae*, the hemichordate *Saccoglossus kowaleskii*, and the cnidarian, *Nematostella vectensis *(Fig. 5). In the case of Msx2 and Msx3, as MEME failed to identify an MH1C domain, we omitted this domain.

This analysis suggests a trend where MH1N and MH1C are most similar to the common ancestor of cnidarians and bilaterians (evolutionary distance = 0.23), and they progressively diverge along the line leading to the common ancestor of vertebrates (evolutionary distance = 0.45–1.59). As a result of this pattern of divergence, MH1N and MH1C remain very similar to each other in the sea anemone *Nematostella*, but they are increasingly distinctive in the hemichordate, the cephalochordate and the mouse/human. This suggests that the MH1N and MH1C domains of cnidarians have evolved in a very conservative fashion since pre-Cambrian times. This conclusion is bolstered by the MEME analysis, which scored both Groucho domains as MH1N domains, whereas in the bilaterian Msx proteins, distinct MH1N and MH1C domains were recognized. Figure 5 also reveals relatively conservative evolution of MH1N and MH1C in non-vertebrate deuterostomes (*Branchiostoma *and *Saccoglossus*) compared to the vertebrate Msx paralogs.

### Conserved synteny of Msx homologs in protostomes, deuterostomes, and cnidarians

In protostomes and deuterostomes, Msx is clustered with other homeobox genes – specifically NK and Tlx genes. Recently, physical linkage between Msx, NK, and Tlx genes was also reported in the genome of the sponge *Amphimedon queenslandica *[8]. Here, we investigated possible physical linkage between Msx, NK, and Tlx genes in the sea anemone, *Nematostella vectensis*, a taxon that is phylogenetically intermediate between sponges and bilaterians. A recent study summarized extensive conserved synteny between *Nematostella *and human, but this study did not identify linkage between Msx, NK, and Tlx in the anemone [79]. We used BLASTx to query a *Nematostella *genome assembly (JGI 1.0; [80]) with all of the homeodomain sequences identified in a previous genome-wide survey [2]. This search localized *Nematostella Msx *to the same 2.38-megabase scaffold (JGI scaffold_06) as *Hmx *(an NK5 ortholog), *NK1*, and *EmxA*. This *Msx *gene is located 17,590 nucleotides from one end of this scaffold. *Msx*, *Hmx*, *Nk1 *reside within 130 kilobases of each other, with *Hmx *located between *Msx *and *Nk1*. *EmxA *lies approximately 1Mb downstream of *Nk1*. Based upon conserved synteny between human and mouse, Holland inferred the existence of an ancestral NK-like cluster that encompassed these same four genes, in addition to other related homeodomain genes [19]. Despite this apparently conserved synteny between human and anemone, when we compared the first 100,000 nucleotides of this anemone scaffold to the non-redundant database at NCBI, we failed to identify any further conserved synteny between human and *Nematostella*. Finally, we searched the *Nematostella *scaffold with human homologs of genes linked to either human *MSX1 *or *MSX2 *using BLASTx but failed to identify any further conserved synteny. Previously, two Msx-like sequences were found in the *Nematostella *genome, between anterior *Antennapedia *type Hox genes (*ax9*, *ax1a*) and another NK type gene (*HLXc-lk*) [81].

### The Nematostella genome has two Groucho loci encoding nearly identical proteins

In taxa such as the sea anemone that possess two MH1 domains that are nearly identical in sequence, it is plausible to expect that they may be functionally redundant. As this domain is implicated in binding the transcriptional repressor Groucho, we sought to investigate whether the diversity of Groucho-binding MH1 domains might mirror the diversity of Groucho genes in the genome. We searched the genome of *Nematostella *for Groucho/TLE homologs. Two distinct genomic scaffolds were identified that contain a *Nematostella Groucho *homolog (see Additional file 5). One of these scaffolds appears to be incompletely sequenced within the *Groucho *gene itself, (Additional file 5 part B). Although the predicted proteins encoded by these two putative *Groucho *genes are nearly identical (only one amino acid difference separates them), the predicted coding regions can be differentiated at many silent sites, and the sizes and sequences of introns are markedly different, suggesting these are in fact two separate loci. *Nematostella Groucho *ESTs present in the NCBI database indicate that both loci are transcribed. Presumably, both Groucho proteins can bind to either MH1N or MH1C domain. These findings suggest that the duplicate MH1 domains within the *Nematostella *Msx protein exhibit functional redundancy.

### Mapping MSX1 mutations by domain

**Figure 6:**
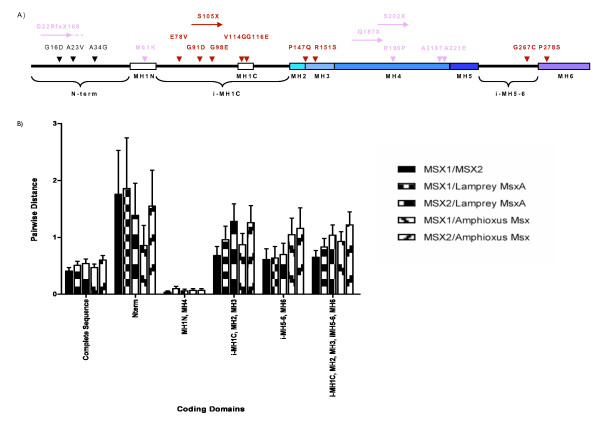
**Human MSX1 domain and mutation map**. A) The positions of disease-associated human mutations are indicated by vertical arrowheads above the domain structure for human MSX1. Missense mutations (e.g., V114G) are described by the wild-type amino acid (e.g., V), the position within the human MSX1 protein (e.g., 114), and the mutation at each site (e.g., G). Nonsense mutations are indicated by horizontal arrows that terminate over the position of the introduced stop codon. Frameshift mutations are indicated by horizontal arrows terminating at the location of the mutation followed by a series of dots. Pink arrowheads denote mutations (M61K, Q187X, S202X, A219T) found in individuals that exhibit an ectodermal dysplasia phenotype. Red arrowheads denote mutations (E78V, G91D, G98E, V114G, G116E, P147Q, R151S, G267C, P278S) found in individuals that exhibit an orofacial cleft phenotype. B) The graph displays pairwise distances between MSX1, MSX2, and two outgroup sequences (*Branchiostoma *Msx and Lamprey MsxA). The lamprey MsxA was compared to MSX1 (small boxes) or MSX2 (large boxes) for each of the domain comparisons. In a similar fashion, *Branchiostoma *Msx was compared to MSX1 (down slanting lines) and MSX2 (up slanting lines).

When all known disease-associated coding mutations previously identified within the human *MSX1 *gene are mapped onto the protein, the mutations causing orofacial clefting (OFC) and the mutations causing ectodermal dysplasias (ED) map to the domain architecture in a non-overlapping fashion (Fig. 6A). OFC mutations, (shown in dark red), [32,38] are found in and around the MH1C, MH3 and MH6 domains, while ED mutations, (shown in light pink), [36,37,82,83] are found within or upstream of MH1N and within MH4 domains.

Because any functional redundancy between MSX1 and MSX2 could mitigate the impact of particular mutations in MSX1, and because MSX1 and MSX2 are unlikely to exhibit functional redundancy in regions where they have undergone extensive sequence divergence, we examined whether the distribution of OFC and ED mutations along the MSX1 protein might be correlated with the degree of divergence between MSX1 and MSX2. Specifically, we compared the distance between MSX1 or MSX2 and two outgroup Msx protein sequences (from lamprey and cephalochordate). Comparisons were first made across the whole protein, and then separate comparisons were performed for five different subregions of the coding sequence (Fig. 6B). Region 1 consists of the N-terminus. Region 2 consists of the MH1N and MH4 domains. Region 3 spans MH3, MH2, MH1c, and the interval between MH1C and MH1N. Region 4 comprises MH6 plus the interval between MH5 and MH6. Finally, region 5 combines regions 3 and 4. Only ED mutations are localized to regions 1 and 2, while OFC mutations are localized to regions 3–5. The pairwise distance data along with standard errors are displayed in Additional file 6, the associated domain definitions for this alignment in Additional file 7 and these data are plotted in Fig. 6B. When we compare homologous segments of human MSX1 and MSX2, the greatest divergence between paralogs is found in the N-terminal segment, and the least divergence is found in the MH1N and MH4 domains. The outgroup comparisons allow us to conclude that MSX2 has generally evolved at a higher rate than MSX1, but this is especially evident within certain regions. For each pairwise comparison, across the whole protein or within particular domains, the evolutionary distance between MSX2 and the sequence from the outgroup taxa is almost always greater than the distance between MSX1 and the outgroup taxa. The lone exception comes when we use the lamprey as the outgroup comparison for the N-terminal region of the protein. In general, the segments that harbor OFC coding mutations in MSX1 are significantly more diverged from MSX2 than those segments that harbor ED coding mutations (Fig. 6B). The frameshift mutation found within the highly variable N-terminal region is the single exception to this pattern (see below for discussion).

### Physiochemical and phylogenetic analysis of protein polymorphisms

**Figure 7:**
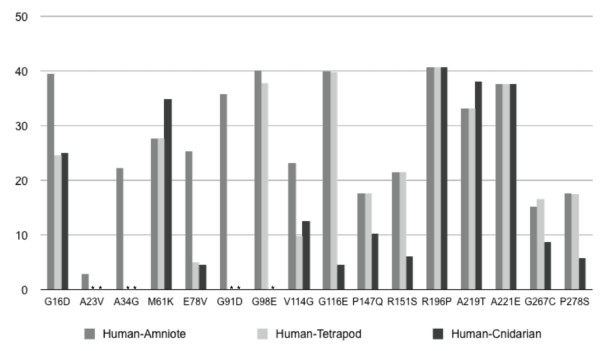
**Physiochemical/phylogenetic analysis of Msx1 mutants**. The MAPP algorithm was used to identify mutations of human Msx1 that appear to violate physiochemical/phylogenetic constraints. Using the full-alignment after subtracting the Msx2, a, d, 3, b and c sequences, positions harboring mutations in human Msx1 were compared with homologous positions in the remaining Msx sequences at progressively more inclusive phylogenetic depths: human-amniote (dark grey bars), human-tetrapod (human plus amniotes and amphibians; light grey bars), human-cnidarian (humans plus amniotes, amphibians, and cnidarians; black bars). * Could not be calculated due to alignment gap(s).

Missense mutations can disrupt the structure of a protein or its intermolecular interactions, and the magnitude of such disruptions (along with their associated phenotypic consequences) can be predicted using either physiochemical or phylogenetic criteria. We used the program MAPP (Multivariate Analysis of Protein Polymorphisms) to evaluate human MSX1 mutations in six different physiochemical dimensions, at three different phylogenetic depths (human (1) inclusive of amniotes, (2) inclusive of tetrapods, and (3) inclusive of cnidarians; Fig. 7; Additional file 8). The higher the MAPP score, the less likely a given mutation is tolerable at the given phylogenetic depth. The analysis reveals that (1) physiochemical considerations alone cannot predict which mutations are likely to be rare on an evolutionary timescale, and (2) the phylogenetic context is critical to evaluating whether a given mutation is likely to be tolerable.

## Discussion

### Early evolution of Msx

**Figure 8:**
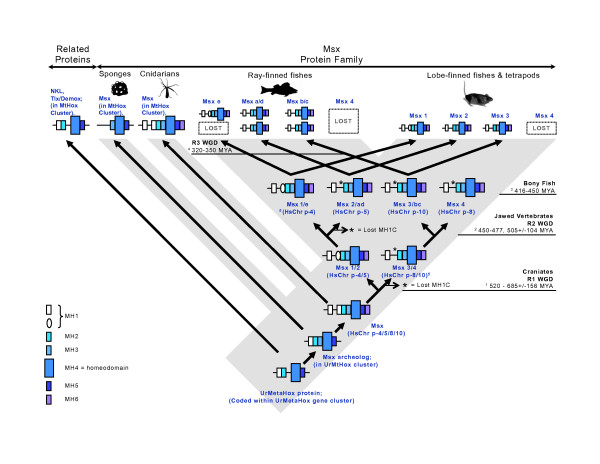
**Msx Domain Evolution Model**. The Msx protein is represented here by a horizontal line overlain by boxes that represent the Msx Homology domains discussed in the text. Presumed loss of the MH1C domain is indicated by asterisks. Inferred ancestral chromosomal segments with homology to human chromosomal paralogons at 4p16, 5q35, 8p and 10q26 are indicated along internal branches. Abbreviations: MYA = Million Years Ago; HsChr p = *Homo sapiens *Chromosome paralogon [86]; MtHox = MetaHox; R1, R2 or R3 = Round 1, 2 or 3; WGD = whole genome duplication. Archeolog = ancestral sequence. Estimated divergence times are taken from the following sources:1 = R1 WGD, origin of craniates = 520 MYA; 687 +/- 156 MYA [85]; 2 = divergence of Chondrichthyes from Osteichthyes = 450 MYA[109,110]. The elephant shark has four Hox clusters, orthologous to known clusters in tetrapods, suggesting Chondrichthyes diverged after the second round of WGD but did not undergo an additional round of duplication as did the osteichthyes. R2 WGD, origin of jawed vertebrates, divergence from agnathans, 477 MYA [111]; 507 +/- 104 MYA, [85]; 3 = divergence of Actinopterygia and Sarcopterygia = 416 MYA [112]; 450 MYA [113]; 4 = R3 WGD within the Actinopterygia = 320 MYA [114]; 350 MYA [113]; 5 = evidence for a 4,5 and 8,10 paralogon split [85,86].

Based on the conserved domain architecture within Msx and MetaHox proteins, the conserved synteny between diverse taxa, and a phylogenetic analysis of amino acid sequences, we can construct a more specific and detailed scenario for the evolution of the Msx family (Fig. 8). Combining all of the domain, phylogenetic, genomic and divergence data above suggests that: 1) Msx likely evolved from an ancestral MetaHox cluster gene, 2) seven ancient domains have been highly conserved over the course of Msx evolution, and 3) the vertebrate Msx paralogs evolved during the two rounds of whole genome duplication, with the MH1C domain either becoming lost or highly modified independently in both the Msx3/b/c and Msx2/d/a lineages.

### Evolution and loss of Msx homology domains

At the time of the cnidarian-bilaterian divergence, there existed a single ancestral *Msx *gene encoding seven distinct Msx Homology (MH) domains including: two Groucho-binding domains (MH1N, MH1C), a Pbx-binding domain (MH2), a linker region (MH3) that includes conserved phosphorylation motifs and a conserved stretch of eight residues adjoining the amino terminus of the homeodomain (MH4), a transcriptional repression domain (MH5), and a PIAS binding domain (MH6). Three of these domains (MH3, MH4, and MH5) are also clearly present in the Msx sequence of the sponge *Amphimedon queenslandica*. Furthermore, as a single Groucho repression domain (MH1), a Pbx binding domain (MH2), a homeodomain (MH4), and a transcriptional repression domain (MH5) are shared with NK, Tlx, and Emx proteins, the domain architecture of Msx points to a common "MetaHox" ancestry for Msx and these other homeobox genes. As there are two MH1 domains in most eumetazoan Msx proteins, zero in the sponge Msx, and one in other MetaHox proteins, we suggest (1) that the MetaHox ancestor possessed one MH1 domain, (2) that the absence of this domain in sponges is due to a secondary loss, and (3) that the MH1 domain duplicated in a eumetazoan Msx gene ancestor prior to the split between cnidarians and bilaterians (Fig. 8).

The shared possession of MH domains across MetaHox proteins could possibly be explained by convergent evolution. For example, Groucho-binding domains are found within a phylogenetically diverse range of metazoan transcription factors, including non-homeobox transcription factors, like Tbox, Fbox, Dorsal [46,58]. These transcription factors are all distantly related, and the shared possession of Groucho domains must reflect convergent evolution at some level. However, a homologous origin of the same basic domain architecture in some ancestral MetaHox gene is suggested by the common domain order within the Msx, NK and Tlx coding sequences, the close phylogenetic relationship of all their homeodomain sequences, and the evidence for ancient clustering of these genes into MetaHox homeobox gene clusters.

### The significance of conserved synteny

The origin of Msx from an ancient MetaHox ancestor is further supported by conserved synteny. In cnidarians (this study) as well as protostomes, deuterostomes, and sponges, Msx is linked to NK, Tlx and Emx genes [8,17-19]. In addition, previous studies on conserved synteny between arthropods and humans, studies that were not specifically focused on homeobox genes, independently revealed that humans and fruit flies share a common ancient chromosomal segment, called a paralogon, that corresponds to modern segments of human chromosome 4p16, 5q35, 2p/8p and 10q26 [84-86]. These very same human genomic regions, at 4p16, 5q35 and 10q26 chromosomal bands, or their syntenic equivalent in the mouse, are the loci for the *Msx1*, *Msx2 *or *Msx3 *genes, respectively. This ancient paralogon shared by arthropods and vertebrates duplicated twice during the two rounds of whole genome duplication that occurred at the base of the vertebrate radiation (Fig. 8; [87]). Importantly, the first genome duplication led to the split between the ancestor of the contemporary chromosome 4/5 paralogons and the ancestor of the contemporary chromosome 8/10 paralogons. This is consistent with the result of our phylogenetic analysis placing *Msx1 *and *Msx2 *as sister lineages because *Msx1 *and *Msx2 *would have derived from a common ancestral sequence on the 4/5 paralogon, into their current positions on human chromosomes 4p16 and 5q35, and would therefore share a closer relationship with each other than with *Msx3*.

The findings described here are largely consistent with an earlier study of partial Msx protein sequences derived mainly from vertebrates [43]. Postlethwait concluded that this phylogenetic analysis was insufficient to convincingly resolve the relationships among the Msx1, Msx2 and Msx3 genes of tetrapods and between the tetrapod and teleost Msx sequences. However, by adding an analysis of human/zebrafish Msx locus synteny, he concluded that zebrafish *msxa *and *msxd *were most likely *Msx2 *orthologs, that *msxe *was an *Msx1 *ortholog and that *msxc *was a *Msx3 *ortholog. He also concluded that human *MSX1 *and *MSX2 *were most likely sister genes on chromosomes 4p and 5q respectively. This was based upon shared orthology of adjacent genes on each chromosome, with the Mouse locus syntenic to human 10q behaving as the outgroup. Finally, he concluded that the zebrafish *msxb *locus shared significant synteny with the human *MSX1 *locus, and therefore that *msxb *is more closely related to *Msx1 *than to *Msx2 *or *Msx3*.

The more extensive phylogenetic analysis and additional analysis of synteny presented here supports all but one of these conclusions: our phylogenetic analysis contradicts the conclusion that teleost *msxb *and tetrapod *Msx1 *are orthologs. Our data instead suggest that the synteny data do not unite *msxb *with *Msx1 *to the exclusion of *Msx3*/*msxc*. For while *msxb *does share synteny with the *Msx1 *locus, it also shares substantial synteny with the *msxc *and *Msx3 *loci. We compared the genomic context of *msxb *and *msxc *in *Danio *and *Fugu *to that of *Msx1 *and *Msx3 *in human and mouse (data not shown). In this analysis, the Msx3/*msxb*/*msxc *genes are united by their closely linked paralogs of the *Adrb3*, *Calcyon*, *Adra1a*, *Taf5*, *Ste20-like Kinase*, *Fgf8/17*, *Adam8 *and *Lbx1 *genes. From this group, only *Calcyon *is shared with the *Msx1*/*msxe*/*Msx2*/*msxd *loci. This finding is consistent with *msxb *being more closely related to *msxc *and *Msx3 *(as our phylogenetic analysis suggests; Fig. 3) than to *msxe *and *Msx1*.

### Evolutionary origins and divergence of vertebrate Msx paralogs

The apparent sister-group relationship between *Msx1 *and *Msx2 *has important implications for the functional evolution of vertebrate Msx proteins. It implies that the MH1C domain was lost twice during vertebrate evolution, once in the common ancestor of the tetrapod-*Msx2*//teleost-*Msxa/d *genes and once in the common ancestor of the tetrapod-*Msx3*//teleost-*Msxb/c *genes (Fig. 8). At some point after the first of two vertebrate whole genome duplication (WGD) events, the MH1C domain was lost from the *Msxb *paralog. This duplication split the ancient paralogon into distinct 4,5 and 8,10 descendants. During the second round of WGD, these two ancestral *Msx *genes were each duplicated again. The duplication of the ancestral 4/5 paralogon created the *Msx1/e *and *Msx2/d/a *lineages. The duplication of the ancestral 8/10 paralogon created the *Msx3/b/c *lineage and presumably another *Msx *locus on the chromosome 8 paralogon ("*Msx4*") that was most likely lost prior to the divergence of the ray-finned fishes (Actinopterygia) and lobe-finned or tetrapod (Sarcopterygia) lineages. Then the remaining three genes, (the *Msx1*, *2 *and *3 *paralogs), were duplicated again during the third round WGD event that took place at the base of the teleosts, (approximately 320 MYA), creating a set of six Msx genes in bony fishes. Subsequently, one of these was lost, presumably the sister paralog of *msxe*, to create the current set of five known Msx genes within teleosts. As explained above, syntenic data from the zebrafish genome are fully compatible with these data; e.g. *msxa *and *msxd *are assigned as orthologs to human *MSX2 *[43]. Sometime after the divergence of primates from rodents, the *Msx3 *gene was lost in the line leading to primates.

The loss of the MH1C domain from Msx2 and Msx3 protein sequence must have important functional consequences because throughout most of animal evolution, the two Groucho-binding domains of Msx1 have been conserved, and they have remained highly similar in sequence. Strong similarity between MH1N and MH1C has been preserved in the Msx proteins of contemporary cnidarians (coral, sea anemone) and non-vertebrate deuterostomes (cephalochordate and hemichordate), and we can infer that it was present in both the ancestral chordate and the last common ancestor of vertebrate Msx paralogs (Fig. 5).

Msx1 and Msx2 have also diverged significantly in the region surrounding the MH6 domain, a region implicated in binding the PIAS protein. Correspondingly, Msx1 and Msx2 have been shown to bind to different paralogs of the PIAS protein family [72]. It is possible that such paralog co-evolution may partially explain the sequence divergence and differing mutation phenotypes of the MH1N and MH1C domains. Takahashi et al., 2008 demonstrated differential binding of a groucho protein, Grg1 to the nearly identical MH1s of *Nematostella *[9]. It remains to be seen if this result represents a positional effect within the anemone Msx protein or whether this reflects differential binding affinities in different groucho paralogs.

Collectively, these data suggest that a process of duplicate gene subfunctionalization, followed by neofunctionalization eventually led to divergent protein functions. The sequence diversification of *Msx1 *and *Msx2 *following their descent from a common ancestral gene has most likely reduced the degree of functional redundancy, which can directly impact the phenotypic consequences of mutations at either locus.

### Mutations

The evolutionary analysis presented here provides a backdrop against which we can evaluate particular coding variants and rate their likelihood of being not merely allelic polymorphisms but disease causing mutations. While identification of mutations among the syndromic, Mendelian disease cases is clear-cut, discriminating true mutations from inconsequential sequence variants in complex disease cases remains controversial, even when supported by genetic data [32-34]. By reconstructing the gain and loss of conserved motifs, and by tracking the diversification of Msx proteins over evolutionary time, we can more easily recognize those human variants that appear incongruous with evolutionarily conserved protein functions. This insight is all the more important since it is probable that complex disease alleles will include weaker mutations that can be difficult to discriminate from background population variants.

Not surprisingly, according to the MAPP analysis, some of the human coding mutations most likely to disrupt critical functions are found at the most conserved positions within the most conserved domains. This is exemplified by the M61K mutation with the MH1N, and the R196P, A219T and A221E mutations within the homeodomain.

However, the analysis also flags mutations that do not reside within conserved domains, such as the G98E mutation, which is found in the region between the highly conserved MH1N and MH1C domains. The MAPP scores for this mutation are uniformly high at all phylogenetic depths examined, suggesting that such a mutation could disrupt some long-conserved function of the protein. Several of the other variants associated with orofacial clefting cases have intermediate MAPP scores perhaps indicative of milder disease alleles.

It is still not possible to definitively decide if the P147Q variant is a weak allele or simply a population-specific variant, as suggested by Tongkobpetch and co-workers [34], and further summarized and evaluated among a large set (5641 individuals) of proband, case family and control individuals [74]. In the latter study, 7 individuals with the P147Q variant displayed a clefting phenotype among a total of 16 carriers with this variant. However, this work identified one family where the P147Q variant did not segregate with the phenotype, again illustrating where the existing genetic data are equivocal. It is interesting that both the P147Q and R151S variants may disrupt potential, conserved phosphorylation motifs within the MH3 domain [74], the region defined as the linker region between the Pbx binding MH2 domain and the homeodomain. Interestingly, among Hox proteins, linker phosphorylation motifs may be deeply conserved [75]. The current data, together with all the previous genetic data, are consistent with the possibility that the P147Q variant, (as well as the E78V variant found amongst Filipino case and control individuals), represents a slightly deleterious allele that was fixed as a result of genetic drift in an initially small effective population [88-90]. Further genetic studies are warranted on these particular alleles.

Although the frameshift mutation that causes selective tooth loss, G22RfsX168, lies within the N-terminal coding segment, this mutation really just represents a complete haploinsufficiency of the protein [91]. This result is consistent with earlier findings that complete haploinsufficiency of MSX1, through mutation at R196P [67] or deletion of one MSX1 gene copy [92] causes ectodermal dysplasia-associated phenotypes, like tooth agenesis. This region also contains three missense mutations found in a high proportion of control samples [32], shown with black arrowheads in Fig. 6. The higher pairwise substitution distances and variability identified within the N-terminal region (Fig. 6B) is consistent with this region being subject to minimal functional constraint, further suggesting that these variants may be coding variants without phenotypic consequences.

The MAPP analysis, like any phylogenetically based analysis, is affected by the inclusion of taxa. Therefore, it comes as no surprise that MAPP comparisons within different phylogenetic contexts produced different scores. In general, variants evaluated by reference to the human-amniote alignments produced the highest MAPP scores, because with less time for divergence, there are fewer substitutions and any change is likely to appear unusual. For example, when the G116E mutation, which resides within MH1C, is compared against the backdrop of amniote or tetrapod Msx1 proteins, its MAPP scores are among the highest. However, the score drops dramatically when that comparison includes cnidarian sequences. This position is almost uniformly conserved back to the base of the tetrapods, being either glycine or a similarly aliphatic residue (Additional file 1 page 4). However in taxa that diverged before the fish-tetrapod split, it is not uncommon to have a negatively charged residue (aspartic acid or glutamic acid) in this position. The tolerance for a negatively charged residue at this position is reflected in Fig. 5, where the vertebrate-cephalochordate ancestor is inferred to have a glutamic acid at this position, the same residue that is found in the corresponding position within MH1N. Interestingly, the inferred residue at this position in the vertebrate ancestor is ambiguous, being either glutamic acid or glycine. However, it appears that since the tetrapods diverged from fish, the MH1C accepts only hydrophobic residues in this region. This is consistent with the evolution of a novel function for the MH1C domain and with the disruption of that function by the G116E substitution.

Another interesting variant, also found within the MH1C domain, is the V114G mutation. The small physiochemical difference between valine and glycine produces only moderately high scores at all depths. However, the MAPP score for this position was one of only three positions that increased substantially when the alignments were made to taxa with deeper divergence times. The deep conservation at this position is reflected in the position-specific sequence matrices (Fig. 2). In both the MH1N and MH1C domains, this valine position exhibits the highest bit score, reflecting its prominent role in the canonical sequence motif for these domains. Since valine is conserved at this position across so many diverse taxa, representing billions of years of cumulative divergence, it is highly likely that the presence of a glycine does represent a real, though perhaps weak disease allele. In summary, this phylogenomic analysis allows these disease associated sequence variants to be quantified and prioritized for future clinical and functional studies.

### Non-random distribution of mutations in Msx1

The nonrandom distribution of mutations for either ectodermal dysplasia or orofacial clefting across the MH domains suggests some unexplained genotype-phenotype correlation. The pairwise distance data (Fig. 6B) reveal that the OFC mutations are localized to regions of the protein that have diverged substantially between MSX1 and MSX2. Of course part of this divergence was the loss of the MH1C domain from MSX2. In addition, in MSX1, MH1C has diverged more than its MH1N since the time after the second whole genome duplication event of vertebrates. Just as different PIAS proteins bind to the MH6 of MSX1 or MSX2, it is possible that different Groucho proteins bind to MH1N and MH1C in MSX1. In tetrapods, paralog coevolution might explain the divergence in sequence and presumably function for the MH1C domain of MSX1. Thus these data are compatible with a model incorporating differential pleiotropy and redundancy of selector protein modules [93], as perhaps exemplified in the present context by the putative PIAS paralog/Msx-MH6 domain co-evolution.

The most likely explanation for the preliminary genotype-phenotype correlation is that those mutations associated with clefting disorders act by a dominant negative mechanism. Clearly the MH1C domain has a discrete function, as it has been conserved in different lineages for billions of years. Our evidence suggests that the MH1C domain may have evolved into a more derived function since the origin of the jawed vertebrates, perhaps involving a co-evolving Groucho paralog. As Msx proteins can form homo and heterodimers with other homeodomains, a missense mutation that disrupts the conserved functions of either MH1C or MH6 could conceivably disrupt the function of additional proteins and manifest itself as a strong dominant negative mutation.

The milder ectodermal dysplasia phenotypes of the MSX1-MH1N and MSX1-MH4 domain mutations can be explained by functional redundancy from the MSX2 domains. As Figure 6 demonstrates that the MH1 and MH4 domains of both MSX1 and MSX2 are highly conserved sequences, this suggests possible functional buffering when MSX1 and MSX2 are co-expressed. Only one published study has reported a mutation in a family with both clefting and ectodermal dysplasia phenotypes [38]. This was a nonsense mutation (S105X) in the coding interval between the MH1N and MH1C domains. Consistent with all the data above, this mutation might be explained by a combination of haploinsufficiency of most of the protein in combination with a dominant negative mechanism acting through the MH1N domain. However all such scenarios remain just speculation until functional studies can shed further light on these possibilities. We must also recognize that the number of mutations reported for MSX1 is still relatively small. In addition, the more minor ectodermal phenotypes may have been under-ascertained in reports concerning the more severe orofacial clefting phenotype.

With the above caveats in mind, the phylogenomic analysis presented here provides a strong intellectual foundation for future in vivo and in vitro functional studies of these mutations. This study may also contribute to diagnostic and preventive interventions wherein such slightly deleterious, complex disease alleles may be overcome by providing an optimal prenatal environment [94-97].

In the future, it may be possible to perform a similar analysis on MSX2 mutations. This is not yet possible as there is a relative dearth of missense MSX2 mutations outside the homeodomain. The collection of human MSX2 mutations (reported on OMIM, *123101) presently consists mostly of loss-of-function mutations (i.e., premature stop codons), homeodomain missense mutations, or frameshift mutations that disrupt/prevent DNA binding and result in parietal foramina (OMIM #168500). There is one gain of function mutation, the Boston type or craniosynostosis type 2 (OMIM #604757), which results from increased homeodomain DNA binding affinity.

### Does the MetaHox cluster represent an animal specific homeobox clade?

In tracing the ancient origin of the Msx Homology coding domains, we found evidence for similar domain architecture in the other descendants of the MetaHox gene cluster. This basic domain architecture could represent a MetaHox synapomorphy, a shared derived trait that unites the Msx, NK, and Tlx genes. This suggests that we can define a monophyletic MetaHox clade comprising Msx, NK, and Tlx genes. In this regard, it is quite encouraging that the NK, Tlx and Demox genes from basal taxa exhibit solid matches to the most conserved MH domains.

The membership of this MetaHox clade may extend to the EHGbox, Hox, and ParaHox genes, if these genes are also descended from a MetaHox ancestor. This possibility is suggested by the presence of Msx, Emx, Tlx, and NK genes and the absence of true Hox and ParaHox genes in the sponges [8]. Similarly, NK and Tlx genes but no Hox or ParaHox genes have been recovered from the ctenophore *Mnemiopsis *[98]. The sponges are widely regarded as the most basal animal phylum, although a recent phylogenomic analysis raises the possibility that ctenophores might be basal to sponges [99]. A MetaHox ancestry of Hox and ParaHox genes is also consistent with recently published homeodomain phylogenies in which Msx, Emx, Tlx are basal to the Hox-ParaHox radiation (e.g., [2]). Finally, physical linkage data from the sponge *Amphimedon *are consistent with NK-like (i.e., MetaHox) genes predating the origin of the *Hox *and *ParaHox *clusters [8].

If Hox and ParaHox genes are derived members of a MetaHox clade, then we should utilize basal MetaHox outgroups to root the evolution of Hox and ParaHox genes. For example, it may be that the evolution of Hox and ParaHox genes was accompanied by the loss of a Groucho-binding domain and other MetaHox domains. Additional evidence of a MetaHox origin of Hox genes comes from an analysis of residues in the PBX binding domain (homologous to MH2) that are characteristic of "anterior" Hox proteins [73]. In anterior Hox proteins, the conserved tryptophan in the PBX-binding domain is preceded by phenylalanine and proline. This is also true of Msx proteins from basal taxa (Additional file 1 page 5). In addition, the residues found at positions 8 and 13 of the Msx homeodomain (F and L, respectively) are functionally important amino acids that again define anterior homeodomain proteins [75]. These shared residues in the PBX-binding domain and homeodomain of Msx and anterior Hox genes suggest a possible evolutionary affinity, wherein primitive anterior Hox genes may have evolved from ancestral Msx genes. This is consistent with the oldest Hox-ParaHox genes being the anterior members of that clade, as recently suggested [81]. As further support for the retention of homologous residues by Msx and anterior Hox proteins, of all the *Amphimedon *sponge homeobox proteins, Msx is most similar to the Hox protein sequences [8].

As the original concept of "MetaHox genes" ([17]; equivalent to the "NK-like genes" independently defined by Pollard and Holland, [61]) did not include EHGbox, Hox, or ParaHox genes, MetaHox as presently defined is a paraphyletic grouping. However, if some urMetaHox gene is the source for the Msx, Tlx, NK, EHGbox, Hox and ParaHox genes of extant animals, we can define an animal-specific MetaHox clade. As meta can mean transforming, this would be an appropriate appellation for these archetypal metazoan developmental genes.

## Conclusion

The results described above all revolve around the role of duplication events on different scales and how these events relate to the subsequent evolution of the taxa involved. On the smallest scale, we provide evidence for the duplication of a Groucho repression domain within an ancient Msx ancestor. This *Msx *gene itself was likely created by the duplication and subsequent divergence of an ancestral urMetaHox gene. Finally, the vertebrate *Msx *gene ancestor was most likely duplicated during the two rounds of whole genome duplication that occurred at the base of the vertebrate radiation.

As with gene duplications, domain duplications can provide a measure of functional redundancy, and such redundancy may facilitate the evolutionary diversification of protein sequences. As Groucho domains are involved in the long-range repression of chromatin, the duplicate Groucho repression domains present in Msx proteins may augment the multimeric assembly of Groucho proteins, and this could be favored by selection, explaining why two highly similar Groucho-binding domains have been conserved over the last 500+ million years of evolution in cnidarians and cephalochordates. However, the subsequent genome and *Msx *gene duplications in vertebrates would have superimposed an additional level of functional redundancy, perhaps facilitated by paralog co-evolution (of the Groucho and PIAS families). This may have allowed for the divergence of the MH1N and MH1C, ultimately resulting in the loss of MH1C in both the Msx2 and Msx3 lineages.

Since duplicate domains and duplicate genes provide two possible layers of functional redundancy, the interpretation of mutations becomes more complex. For example, several scenarios could explain the finding that mutations in the MH1N and MH1C domains of MSX1 result in different human disease phenotypes. This could reflect the fact that these domains have undergone a significant functional divergence. However, this could also reflect partial functional overlap between Msx paralogs, such that Msx2 may compensate for mutations in Msx1 where these two proteins share a domain (e.g., MH1N), but it may be unable to compensate for mutations in a domain of Msx1 that is lacking in Msx2. While the details remain to be worked out, these data in total suggest that duplicate protein domains may provide the initial redundancy that allows the evolution of coding domain subfunctions, even within a vitally important, pleiotropically expressed regulatory gene like *Msx1*. These results also raise the fascinating possibility that the divergence of MH domains within vertebrates might be responsible for vertebrate specific developmental trajectories aligned with alternate chromatin states.

## Methods

### Sequence retrieval

We obtained predicted amino acid sequences for 46 Msx proteins from representative poriferans, cnidarians, protostomes, and deuterostomes. To represent cnidarians, we selected Msx sequences from a sea anemone (*Nematostella vectensis*, BAG11598) and a coral (*Acropora millepora*; ABK41269). Single Msx proteins were retrieved from one lophotrochozoan protostome (*Platynereis*; CAJ38810), three non-vertebrate deuterostomes (*Branchiostoma *[ABD97280], *Saccoglossus *[ABD97280], and *Heliocidaris *[AAY86178]) and one jawless craniate *Petromyzon *[ABW76121]. Msx1, Msx2 and Msx3 paralogs of tetrapod vertebrates were retrieved from mammals (*Homo *[AAH67353, NP_002440], *Pan *[AAZ30465, ABM92019], *Macaca *[AAZ30466], *Monodelphis *[XP_001364443, XP_001378128], *Mus *[AAB35456, Q03358, AAC15459], *Rattus *[NP_112321, NP_037114, BAE92723], *Bos *[AAI20209], *Canis *[XP_001370688, CAC37368]), birds (*Coturnix *[P23410], *Gallus *[P28361, P28362]), amphibians (*Ambystoma *[AAS17879, BAD07299], *Eleutherodactylus *[AAS98252, AAS98253], *Notophthalmus *[AAI41725], *Xenopus *[AAH62514, AAH81101, AAH64202, NP_001032329, NP_571348]), bony fishes (*Danio *[Msxa: NP_571349; Msxb: NP_571335; Msxc: NP 571347; Msxd: GENSCAN00000023921; Msxe: NP_571348;], *Fugu *[Msxb: GENSCAN00000028575; Fr Msxc: GENSCAN 00000022367 plus conceptual translation to complete; Msxd: GENSCAN 00000008872 plus conceptual translation to complete; Msxe: GENSCAN 00000010652, plus conceptual translation to complete;], *Tetraodon *[Msxc: GIDT00016399001; Msxd: GIDT00024806001; Msxe: CAG01864), and cartilaginous fishes (*Scyliorhinus *[BAE98267]). The demosponge Msx sequence was taken from the recent publication of Larroux et al., 2007 [8].

To examine whether the protein coding domains identified within a small set of Msx protein sequences were generally more conserved outside of the Msx family, a hidden Markov model was created using MetaMEME and subsequently tested upon a set of twenty-three sequences that included several Msx protein sequences and all the known full-length poriferan NK and Tlx genes (Additional file 2); HsMsx1, NP 002439.2 *Homo sapiens*; StMsx, BAE98267 *Scyliorhinus torazame*; AcmMsx3, ABK41269 *Acropora millepora*; NevMsx1, BAG11598 *Nematostella vectensis*; AmqMsx *Amphimedon queenslandica*; EflMsx AAA20151 *Ephydatia fluviatilis*; AmqBshL, ACA04743 *Amphimedon queenslandica*; NevNK1, NK1 *Nematostella vectensis*; PdTlx, ABQ10643 *Platynereis dumerilii*; PdNK1, CAJ38797 *Platynereis dumerilii*; AmqNK2-3-4L, ACA04745 *Amphimedon queenslandica*; SbNK2-3-4L, CAD37942 *Suberites domuncula*; AmqBarH, BarH *Amphimedon queenslandica*; AmqTlxLProx2, ACA04744 *Amphimedon queenslandica*; EflNK2L, AAA20149 *Ephydatia fluviatilis*; PsDemox, AAX77088 *Potamolepis *sp.; EmEmH-3, AAC18965 *Ephydatia muelleri*; BiDemox, AAX77090 *Baikalospongia intermedia*; SdHoxa1, CAD37941 *Suberites domuncula*; EflEmH-3, AAB04117 *Ephydatia fluviatilis*; SlEmH-3, AAP75575 *Spongilla lacustris*; ThEmH-3, AAP75576 *Trochospongilla horrida*; EfrEmH-3, AAP75574 *Eunapius fragilis*.

### Protein domain identification

Conserved protein domains were identified using MEME (Multiple Expectation Maximization for Motif Elicitation; ; [100]). As the selection of taxa can bias the identification of domains, we began by comparing a small set of deeply diverged taxa in order to avoid over-weighting lineage-specific protein features; we compared human MSX1 with the only known Msx proteins from a cephalochordate, a tunicate, an echinoderm, a polychaete worm, and a coral. We subsequently compared a broad selection of Msx paralogs from tetrapods and fishes along with single Msx proteins from non-vertebrate deuterostomes (cephalochordate, tunicate, hemichordate, echinoderm) and a polychaete annelid. Parameter settings within MEME were as follows: occurrences of a single motif = any number; minimum length of a motif = 8 amino acids; maximum length of a motif = 68. Setting the maximum domain length to 68 identified as a single domain a region spanning the canonical 60-amino acid homeodomain plus eight highly conserved amino acids immediately upstream of the homeodomain, the so-called "N8" domain. This setting gave the smallest domain that included the homeodomain. Once domain patterns were identified by MEME, we then searched online protein databases for similar motif patterns via a Hidden Markov Model based approach using the program MetaMEME [101].

### Multisequence alignments

Msx protein sequences were aligned using the Clustal alignment tool found within the MEGA 4.0 program under the default settings [76]. This was accomplished in several reproducible steps. First, a master alignment was generated using the Msx sequences from two cnidarians and two non-vertebrate deuterostomes (*Nematostella*, *Acropora*, *Saccoglossus *and *Branchiostoma*). In order that the motifs identified by MEME remain in register, the master alignment was assembled in three separate blocks. The first block encompassed all of the residues from the start methionine to the highly conserved phenylalanine in the MH1N motif. The second block extended from the same highly conserved phenylalanine in MH1N to the conserved tryptophan in MH2 (the hexapeptide). The third block extended from the tryptophan in MH2 to the end of the protein. Each of these blocks was separately aligned with Clustal using the default parameters. The resulting master alignment maintained all of the conserved motifs in register. The other MSX sequences were then aligned to the master alignment, once again in three separate blocks. The highly divergent MSX sequences from the clamworm *Platynereis *and the sea urchin *Heliocidaris *were excluded from the alignment because their presence proved highly disruptive. We also produced a gap-free alignment by eliminating all positions harboring alignment gaps in the master alignment. Both the master alignment (Additional File 1 page1-8) and the gap-free alignment (Additional File 4 page 1-2) were used in the phylogenetic analyses.

### Estimating the best model of amino acid substitution

The best model of the amino acid substitution process was chosen from among 80 possible models using the program ProtTest 1.3 [102] for both the full alignment and the gap-free alignment. The substitution process was estimated simultaneously along with the tree topology and branch lengths. For both the full alignment and the gap-free alignment, the empirically determined JTT substitution matrix [103] outperformed other substitution matrices, and incorporating rate variation among sites significantly improved the model (gamma distribution of rate variation among sites, α = 1.2).

### Reconstructing the evolution of motifs MH1N and MH1C

The ancestral sequences of MH1N and MH1C were inferred for four key ancestors (the vertebrate ancestor, the vertebrate-cephalochordate ancestor, the chordate-hemichordate ancestor, and the cnidarian-bilaterian ancestor) based on sequences found in five extant animals: *Homo*, *Mus*, *Branchiostoma*, *Saccoglossus*, and *Nematostella*. To facilitate comparisons between MH1N and MH1C, only the conserved core of the domain was used (FSVXXXXXX). In all but two instances, the sequences used for the extant animals were taken directly from the alignment (see Additional file 1). However, in the case of MH1N from mouse Msx3 and MH1C from *Nematostella *Msx, single alignment gaps were removed. Ancestral character states were inferred using MacClade (version 4.03). Where the ancestral character state was ambiguous, all plausible character states identified by the program were considered in subsequent distance calculations. For both MH1N and MH1C, evolutionary distances were calculated from each ancestor to its descendent(s) using the JTT distance matrix. Where MacClade inferred multiple possible ancestral states, the range of possible evolutionary distances were calculated. In addition, the evolutionary distance between MH1N and MH1C was calculated for each ancestor and each extant animal. Distance calculations were not made to MH1C in Msx2 and Msx3 because no significant match to the MH1C motif was identified in these proteins.

### Evolutionary pairwise distance calculation

Using MEGA (v. 4.0), pairwise evolutionary distances were calculated between human MSX1 and MSX2, shark Msx1, Lamprey MsxA (ABW76121) and the single Msx from Amphioxus (Additional file 6). Individual domains defined by MEME were delineated within MEGA, so distance calculations could be made on a domain-by-domain basis (Additional file 7). After the deletion of all alignment gaps, each pairwise distance was calculated from 1000 bootstrapped datasets using the JTT substitution matrix, assuming homogenous rates among lineages. Rate variation among sites was assumed to be different, with the gamma parameter set to 1.00. Results were graphed with GraphPad Prism 5 software (GraphPad Software, La Jolla, CA).

### Phylogenetic analysis

The evolutionary relationships among 44 Msx class genes were estimated by neighbor-joining [104] using the computer package Phylip (version 3.6.1; [105]). Distances among homeodomains were calculated using the ProtDist program of Phylip and the James-Taylor-Thorton (JTT) distance matrix [103]. Support for clades on the neighbor-joining tree was assessed by 1000-replicates of the bootstrap [106]. The tree was re-drawn and re-rooted using the cnidarian sequences as an outgroup with the computer program MacClade, version 4.03 [107].

The three possible topologies relating vertebrate Msx1, Msx2, and Msx3 were explicitly compared using a smaller dataset. The ingroup consisted of Msx1, Msx2, and Msx3 proteins from *Mus*, *Rattus*, and *Monodelphis*, the only taxa for which all three paralogs were represented. The outgroup consisted of the single Msx sequences from the sea anemone, *Nematostella*, and the lancelet, *Branchiostoma*. Both neighbor-joining and maximum-likelihood trees were constructed for these eleven taxa using the full-length alignment. Distances between sequences were calculated using the JTT matrix, with and without rate variation among sites (the gamma parameter was set to 1.2). The support for alternate hypotheses was evaluated using 1000 replicates of the bootstrap [106]. Maximum likelihood analyses were also performed on the same dataset, once again using the JTT substitution matrix, with and without rate variation among sites. Support for alternate hypotheses was evaluated by (1) comparing the likelihood of the alternate topologies and (2) comparing the bootstrap support for each of the three possible pairings of Msx1, Msx2 and Msx3.

### Evaluation of physiochemical changes in amino acid variants

The Multivariate Analysis of Protein Polymorphism program (MAPP) found online at: , was used to evaluate the physiochemical disruption of amino acid substitutions and the tolerance for a particular amino acid at a particular position within Msx1 proteins over evolutionary time [108]. The alignments and tree topologies with branch lengths utilized in this analysis were derived from the Msx1, Msxe and prevertebrate Msx1 ortholog full sequence alignment, as described above. Those sites labeled with N/A in Additional file 8 had no MAPP score because of too many gaps in the alignment.

## Authors' contributions

JF performed or guided all the phylogenetic analyses, contributed the relevant text and contributed extensively to the writing of the manuscript. MM performed the *Nematostella *genome searches for synteny of its Msx locus to that of other taxa, mapped the two *Nematostella *Groucho loci and wrote the text for those sections. PJ first conceived of these ideas and relationships during his Ph.D. thesis work at the University of Iowa. He also wrote most of the initial text for the project and performed the MEME, metaMEME and MAST analysis as well as the MEGA pairwise distance analysis and provided the clinical focus. The final text went through many revisions with input from all the authors.

## Supplementary Material

Additional file 1 **Msx Alignment (full alignment).** This file represents the multisequence alignment of Msx protein sequences from 44 taxa, as described in the text. Click here for file

Additional file 2 **Hidden Markov Model MetaMEME output.** This file displays a sample of MetaMEME scores and alignments evaluated against a Hidden Markov Model trained on diverse Msx protein sequences. Click here for file

Additional file 3 **Msx Phylogeny (without gaps).** This file represents the phylogenetic analysis of the ungapped alignment, as described in the text. Click here for file

Additional file 4 **Msx Alignment (without gaps).** This file represents the multisequence alignment of Msx protein sequences after all gaps were removed, as described in the text. Click here for file

Additional file 5 **Nematostella Groucho Loci with Exon Structure.** This file illustrates the exon/intron map for the Nematostella Groucho1 and Groucho1a genes and their correspondence to known Nematostella ESTs. Click here for file

Additional file 6 **Pairwise Evolutionary Distance Calculations for *MSX1 *and *MSX2 *compared to Shark Msx1, Lamprey MsxA and Amphioxus Msx.** This file displays the pairwise evolutionary distance calculations of Msx domain subsets against different outgroup sequences. Click here for file

Additional file 7 **Msx Domain definition alignments for Pairwise Distance Calculations.** This file displays the domain definitions used in the Pairwise Distance Calculations. Click here for file

Additional file 8 **Table of MAPP scores for Msx1 alignments inclusive of Human to Cnidarians, Tetrapods or Amniotes.** This file displays the Multivariate Analysis of Protein Polymorphism (MAPP) Scores of different known human MSX1 missense coding variants within different phylogenetic depths. Click here for file
