# Supplementary material for: Domain duplication, divergence, and loss events in vertebrate Msx paralogs reveal phylogenomically informed disease markers
Source: BMC Evol Biol. 2009 Jan 20;9:18. doi: 10.1186/1471-2148-9-18 (PMC2655272; doi:10.1186/1471-2148-9-18)
Supplement: Additional file 1 — Msx Alignment (full alignment). This file represents the multisequence alignment of Msx protein sequences from 44 taxa, as described in the text. [file 1471-2148-9-18-S1.pdf]

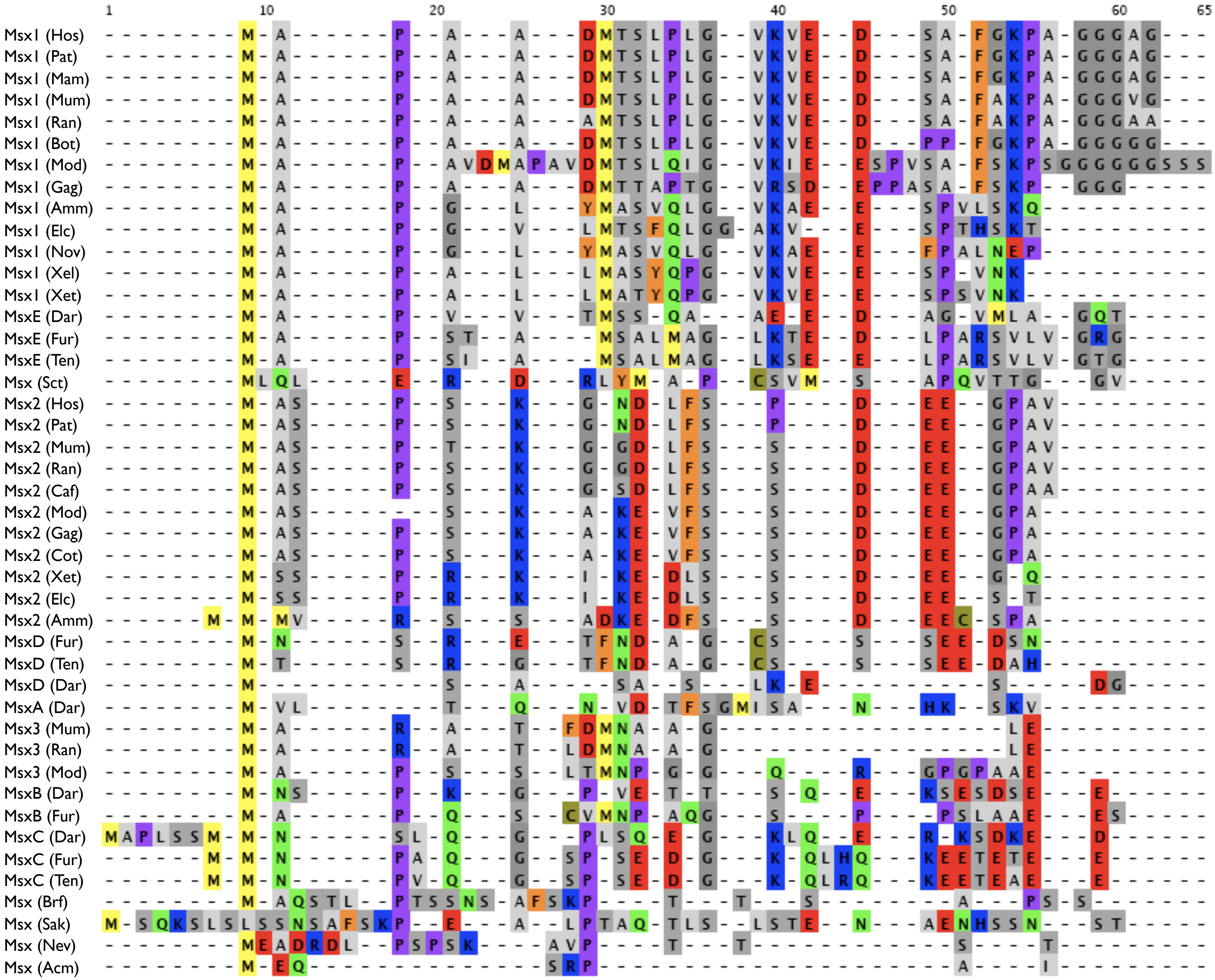
1
10
20
30
40
50
60
65
Msx1 (Hos)
Msx1 (Pat)
Msx1 (Mam)
Msx1 (Mum)
Msx1 (Ran)
Msx1 (Bot)
Msx1 (Mod)
Msx1 (Gag)
Msx1 (Amm)
Msx1 (Elc)
Msx1 (Nov)
Msx1 (Xel)
Msx1 (Xet)
MsxE (Dar)
MsxE (Fur)
MsxE (Ten)
Msx (Sct)
Msx2 (Hos)
Msx2 (Pat)
Msx2 (Mum)
Msx2 (Ran)
Msx2 (Caf)
Msx2 (Mod)
Msx2 (Gag)
Msx2 (Cot)
Msx2 (Xet)
Msx2 (Elc)
Msx2 (Amm)
MsxD (Fur)
MsxD (Ten)
MsxD (Dar)
MsxA (Dar)
Msx3 (Mum)
Msx3 (Ran)
Msx3 (Mod)
MsxB (Dar)
MsxB (Fur)
MsxC (Dar)
MsxC (Fur)
MsxC (Ten)
Msx (Brf)
Msx (Sak)
Msx (Nev)
Msx (Acm)

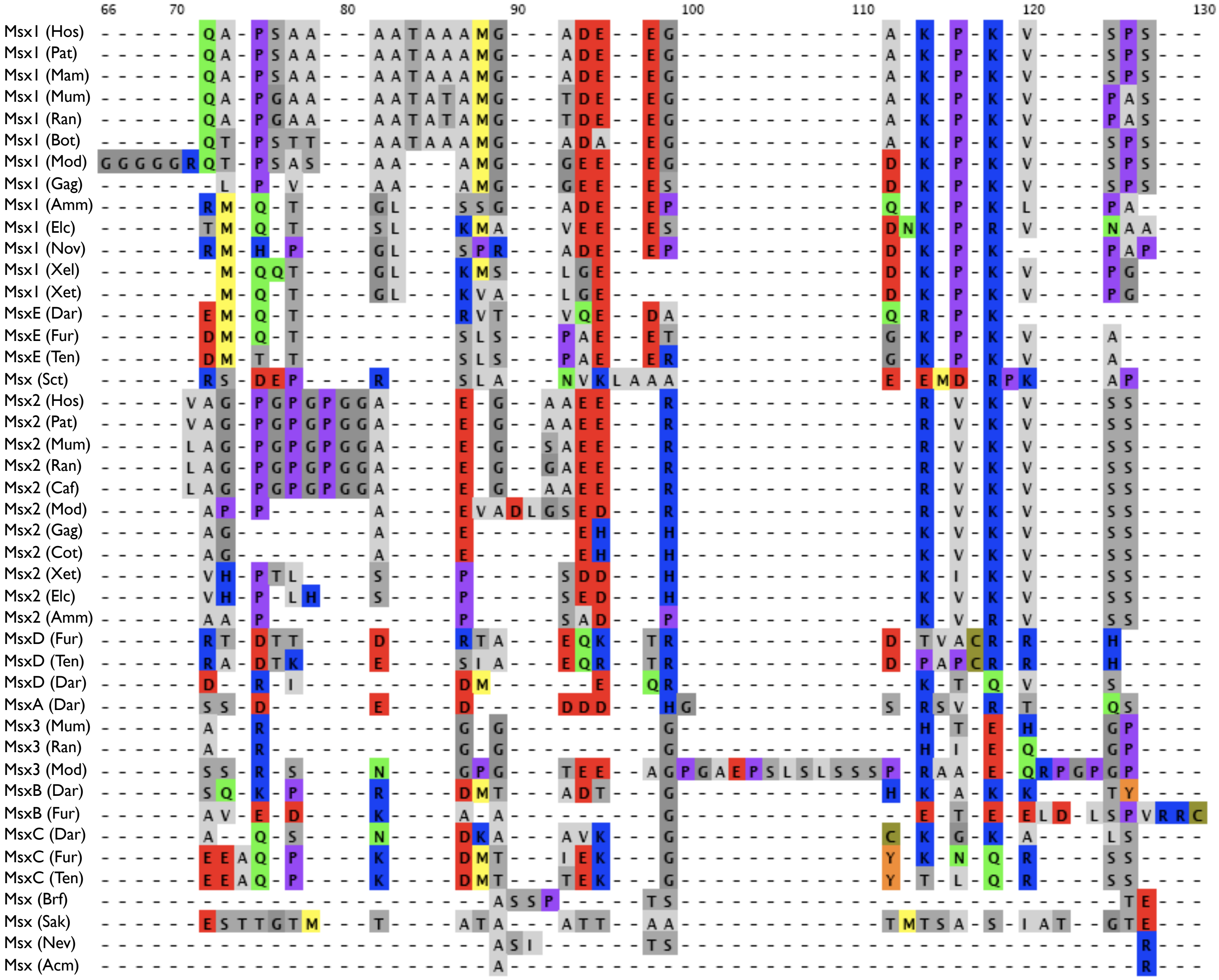
66
70
80
90
100
110
120
130
Msx1 (Hos)
Msx1 (Pat)
Msx1 (Mam)
Msx1 (Mum)
Msx1 (Ran)
Msx1 (Bot)
Msx1 (Mod)
Msx1 (Gag)
Msx1 (Amm)
Msx1 (Elc)
Msx1 (Nov)
Msx1 (Xel)
Msx1 (Xet)
MsxE (Dar)
MsxE (Fur)
MsxE (Ten)
Msx (Sct)
Msx2 (Hos)
Msx2 (Pat)
Msx2 (Mum)
Msx2 (Ran)
Msx2 (Caf)
Msx2 (Mod)
Msx2 (Gag)
Msx2 (Cot)
Msx2 (Xet)
Msx2 (Elc)
Msx2 (Amm)
MsxD (Fur)
MsxD (Ten)
MsxD (Dar)
MsxA (Dar)
Msx3 (Mum)
Msx3 (Ran)
Msx3 (Mod)
MsxB (Dar)
MsxB (Fur)
MsxC (Dar)
MsxC (Fur)
MsxC (Ten)
Msx (Brf)
Msx (Sak)
Msx (Nev)
Msx (Acm)

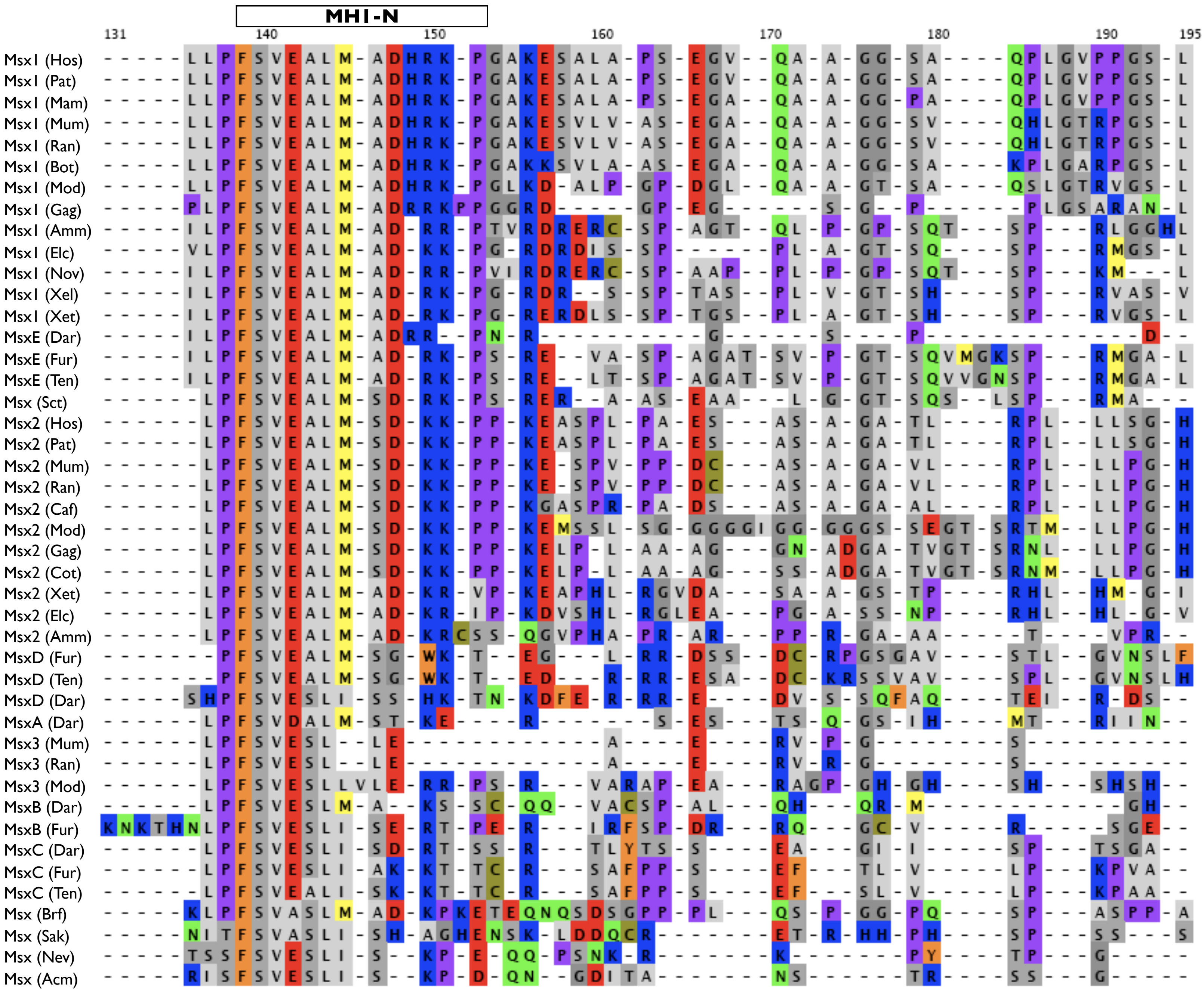

MH1-N
131
140
150
160
170
180
190
195
Msx1 (Hos)
Msx1 (Pat)
Msx1 (Mam)
Msx1 (Mum)
Msx1 (Ran)
Msx1 (Bot)
Msx1 (Mod)
Msx1 (Gag)
Msx1 (Amm)
Msx1 (Elc)
Msx1 (Nov)
Msx1 (Xel)
Msx1 (Xet)
MsxE (Dar)
MsxE (Fur)
MsxE (Ten)
Msx (Sct)
Msx2 (Hos)
Msx2 (Pat)
Msx2 (Mum)
Msx2 (Ran)
Msx2 (Caf)
Msx2 (Mod)
Msx2 (Gag)
Msx2 (Cot)
Msx2 (Xet)
Msx2 (Elc)
Msx2 (Amm)
MsxD (Fur)
MsxD (Ten)
MsxD (Dar)
MsxA (Dar)
Msx3 (Mum)
Msx3 (Ran)
Msx3 (Mod)
MsxB (Dar)
MsxB (Fur)
MsxC (Dar)
MsxC (Fur)
MsxC (Ten)
Msx (Brf)
Msx (Sak)
Msx (Nev)
Msx (Acm)

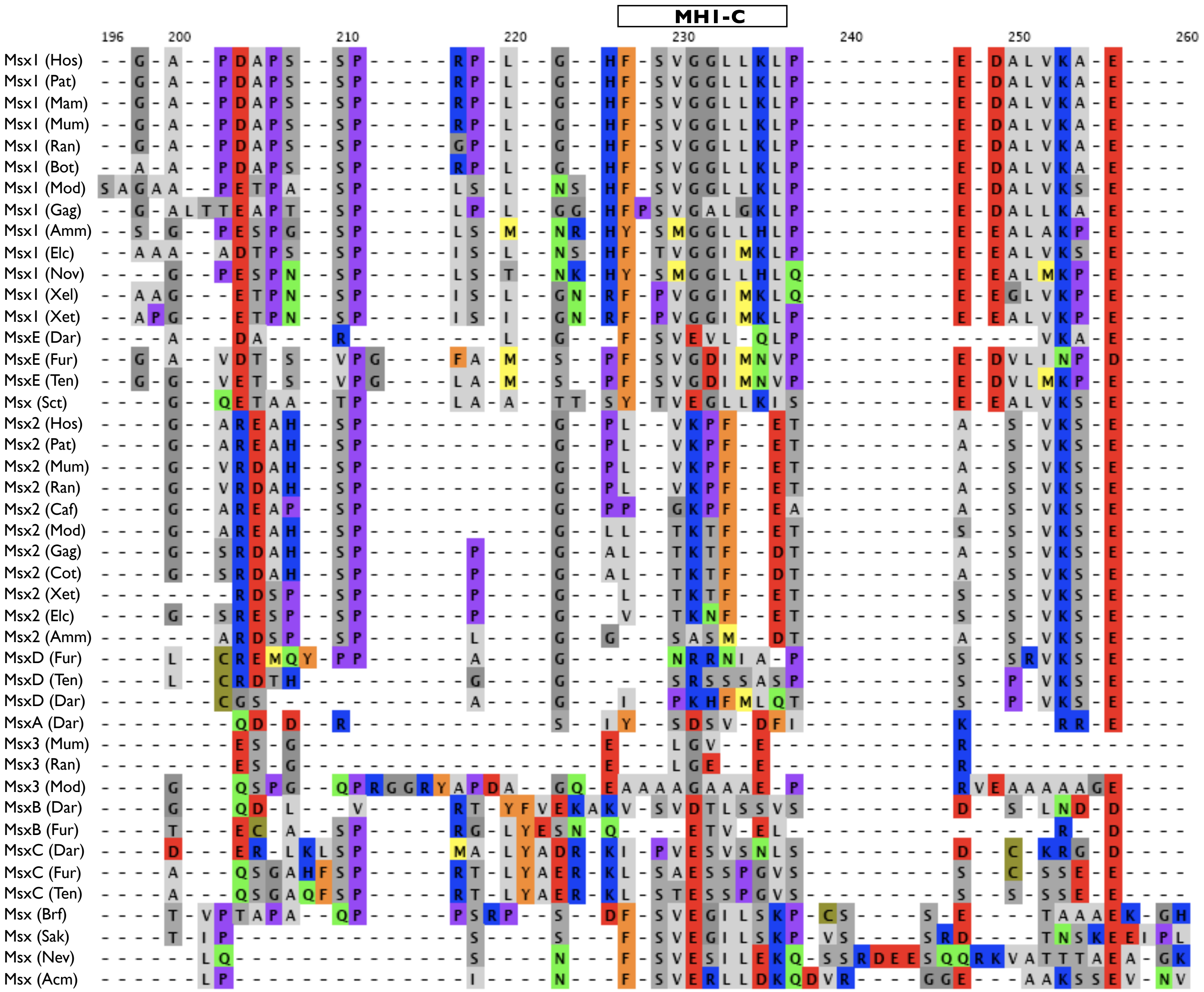

MH1-C
196
200
210
220
230
240
250
260
Msx1 (Hos)
Msx1 (Pat)
Msx1 (Mam)
Msx1 (Mum)
Msx1 (Ran)
Msx1 (Bot)
Msx1 (Mod)
Msx1 (Gag)
Msx1 (Amm)
Msx1 (Elc)
Msx1 (Nov)
Msx1 (Xel)
Msx1 (Xet)
MsxE (Dar)
MsxE (Fur)
MsxE (Ten)
Msx (Sct)
Msx2 (Hos)
Msx2 (Pat)
Msx2 (Mum)
Msx2 (Ran)
Msx2 (Caf)
Msx2 (Mod)
Msx2 (Gag)
Msx2 (Cot)
Msx2 (Xet)
Msx2 (Elc)
Msx2 (Amm)
MsxD (Fur)
MsxD (Ten)
MsxD (Dar)
MsxA (Dar)
Msx3 (Mum)
Msx3 (Ran)
Msx3 (Mod)
MsxB (Dar)
MsxB (Fur)
MsxC (Dar)
MsxC (Fur)
MsxC (Ten)
Msx (Brf)
Msx (Sak)
Msx (Nev)
Msx (Acm)

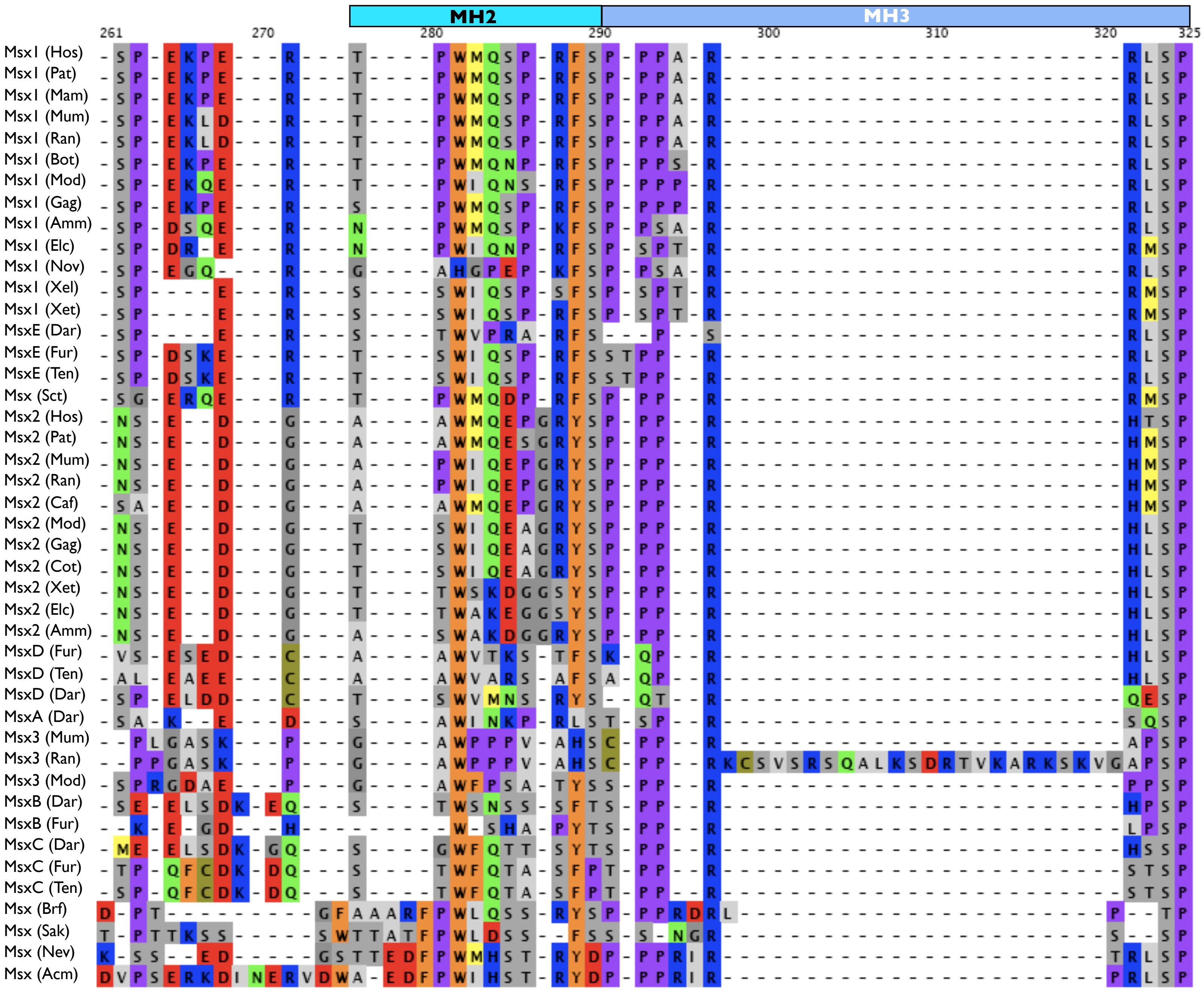
MH2
MH3
261
270
280
290
300
310
320
325
MsxI (Hos)
MsxI (Pat)
MsxI (Mam)
MsxI (Mum)
MsxI (Ran)
MsxI (Bot)
MsxI (Mod)
MsxI (Gag)
MsxI (Amm)
MsxI (Elc)
MsxI (Nov)
MsxI (Xel)
MsxI (Xet)
MsxE (Dar)
MsxE (Fur)
MsxE (Ten)
Msx (Sct)
Msx2 (Hos)
Msx2 (Pat)
Msx2 (Mum)
Msx2 (Ran)
Msx2 (Caf)
Msx2 (Mod)
Msx2 (Gag)
Msx2 (Cot)
Msx2 (Xet)
Msx2 (Elc)
Msx2 (Amm)
MsxD (Fur)
MsxD (Ten)
MsxD (Dar)
MsxA (Dar)
Msx3 (Mum)
Msx3 (Ran)
Msx3 (Mod)
MsxB (Dar)
MsxB (Fur)
MsxC (Dar)
MsxC (Fur)
MsxC (Ten)
Msx (Brf)
Msx (Sak)
Msx (Nev)
Msx (Acm)

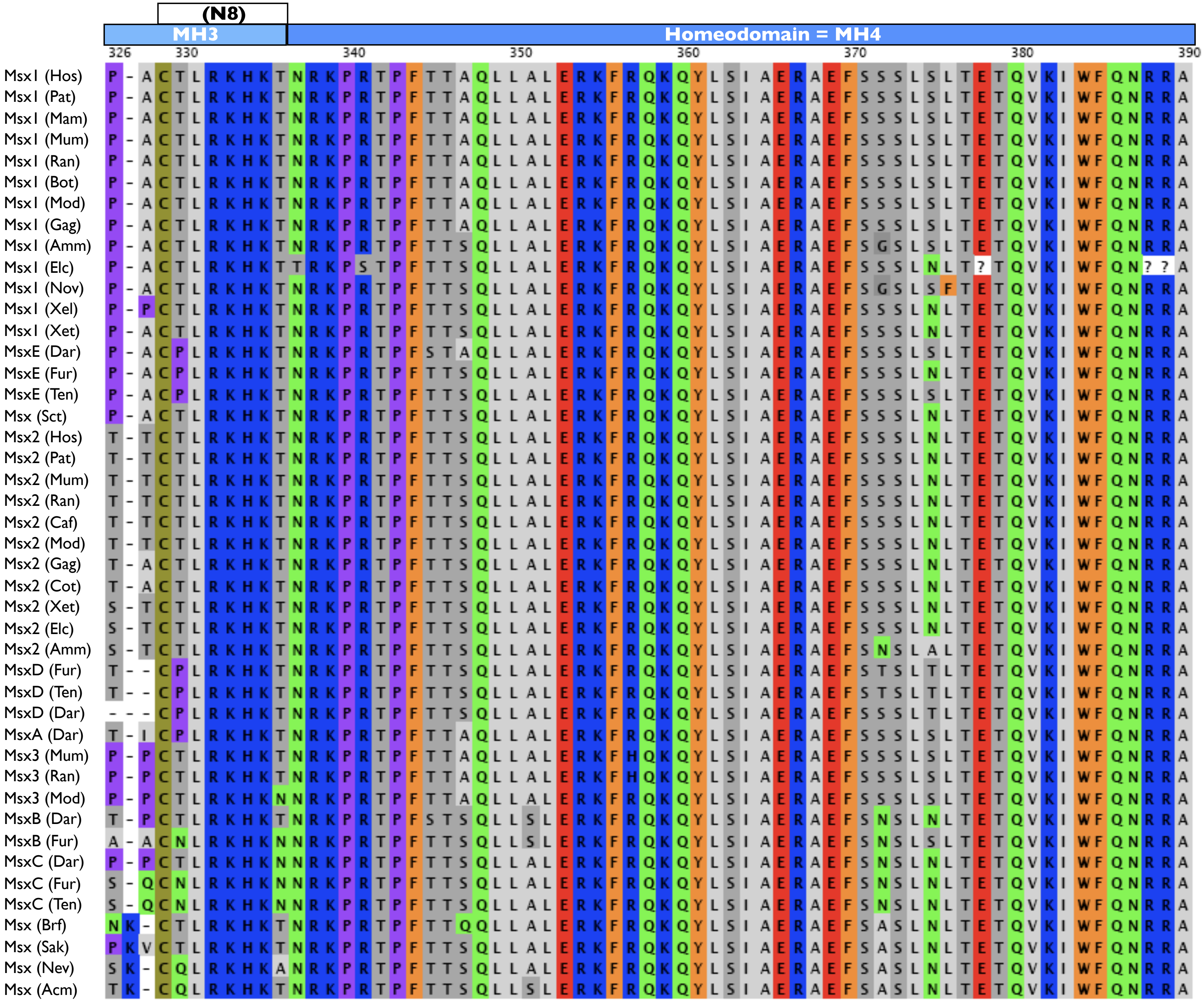

(N8)
MH3
Homeodomain = MH4
326 330 340 350 360 370 380 390
Msx1 (Hos) P-ACTLRKHKTNRKPRTPFTTAQLLALERKFRQKQYLSIAERAEFSSSLSLTETQVKIWFQNRRA
Msx1 (Pat) P-ACTLRKHKTNRKPRTPFTTAQLLALERKFRQKQYLSIAERAEFSSSLSLTETQVKIWFQNRRA
Msx1 (Mam) P-ACTLRKHKTNRKPRTPFTTAQLLALERKFRQKQYLSIAERAEFSSSLSLTETQVKIWFQNRRA
Msx1 (Mum) P-ACTLRKHKTNRKPRTPFTTAQLLALERKFRQKQYLSIAERAEFSSSLSLTETQVKIWFQNRRA
Msx1 (Ran) P-ACTLRKHKTNRKPRTPFTTAQLLALERKFRQKQYLSIAERAEFSSSLSLTETQVKIWFQNRRA
Msx1 (Bot) P-ACTLRKHKTNRKPRTPFTTAQLLALERKFRQKQYLSIAERAEFSSSLSLTETQVKIWFQNRRA
Msx1 (Mod) P-ACTLRKHKTNRKPRTPFTTAQLLALERKFRQKQYLSIAERAEFSSSLSLTETQVKIWFQNRRA
Msx1 (Gag) P-ACTLRKHKTNRKPRTPFTTAQLLALERKFRQKQYLSIAERAEFSSSLSLTETQVKIWFQNRRA
Msx1 (Amm) P-ACTLRKHKTNRKPRTPFTTSQLLALERKFRQKQYLSIAERAEFSGSLSLTETQVKIWFQNRRA
Msx1 (Elc) P-ACTLRKHKTTRKPSTPFTTSQLLALERKFRQKQYLSIAERAEFSSSLNLT?TQVKIWFQN??A
Msx1 (Nov) P-ACTLRKHKTNRKPRTPFTTSQLLALERKFRQKQYLSIAERAEFSGSLSFTETQVKIWFQNRRA
Msx1 (Xel) P-PCTLRKHKTNRKPRTPFTTSQLLALERKFRQKQYLSIAERAEFSSSLNLTETQVKIWFQNRRA
Msx1 (Xet) P-ACTLRKHKTNRKPRTPFTTSQLLALERKFRQKQYLSIAERAEFSSSLNLTETQVKIWFQNRRA
MsxE (Dar) P-ACPLRKHKTNRKPRTPFSTAQLLALERKFRQKQYLSIAERAEFSSSLSLTETQVKIWFQNRRA
MsxE (Fur) P-ACPLRKHKTNRKPRTPFTTSQLLALERKFRQKQYLSIAERAEFSSSLNLTETQVKIWFQNRRA
MsxE (Ten) P-ACPLRKHKTNRKPRTPFTTSQLLALERKFRQKQYLSIAERAEFSSSLSLTETQVKIWFQNRRA
Msx (Sct) P-ACTLRKHKTNRKPRTPFTTSQLLALERKFRQKQYLSIAERAEFSSSLNLTETQVKIWFQNRRA
Msx2 (Hos) T-TCTLRKHKTNRKPRTPFTTSQLLALERKFRQKQYLSIAERAEFSSSLNLTETQVKIWFQNRRA
Msx2 (Pat) T-TCTLRKHKTNRKPRTPFTTSQLLALERKFRQKQYLSIAERAEFSSSLNLTETQVKIWFQNRRA
Msx2 (Mum) T-TCTLRKHKTNRKPRTPFTTSQLLALERKFRQKQYLSIAERAEFSSSLNLTETQVKIWFQNRRA
Msx2 (Ran) T-TCTLRKHKTNRKPRTPFTTSQLLALERKFRQKQYLSIAERAEFSSSLNLTETQVKIWFQNRRA
Msx2 (Caf) T-TCTLRKHKTNRKPRTPFTTSQLLALERKFRQKQYLSIAERAEFSSSLNLTETQVKIWFQNRRA
Msx2 (Mod) T-TCTLRKHKTNRKPRTPFTTSQLLALERKFRQKQYLSIAERAEFSSSLNLTETQVKIWFQNRRA
Msx2 (Gag) T-ACTLRKHKTNRKPRTPFTTSQLLALERKFRQKQYLSIAERAEFSSSLNLTETQVKIWFQNRRA
Msx2 (Cot) T-ACTLRKHKTNRKPRTPFTTSQLLALERKFRQKQYLSIAERAEFSSSLNLTETQVKIWFQNRRA
Msx2 (Xet) S-TCTLRKHKTNRKPRTPFTTSQLLALERKFRQKQYLSIAERAEFSSSLNLTETQVKIWFQNRRA
Msx2 (Elc) S-TCTLRKHKTNRKPRTPFTTSQLLALERKFRQKQYLSIAERAEFSSSLNLTETQVKIWFQNRRA
Msx2 (Amm) S-TCTLRKHKTNRKPRTPFTTSQLLALERKFRQKQYLSIAERAEFSNSLALTETQVKIWFQNRRA
MsxD (Fur) T--CPLRKHKTNRKPRTPFTTSQLLALERKFRQKQYLSIAERAEFSTSLTLTETQVKIWFQNRRA
MsxD (Ten) T--CPLRKHKTNRKPRTPFTTSQLLALERKFRQKQYLSIAERAEFSTSLTLTETQVKIWFQNRRA
MsxD (Dar) ---CPLRKHKTNRKPRTPFTTSQLLALERKFRQKQYLSIAERAEFSSSLTLTETQVKIWFQNRRA
MsxA (Dar) T-ICPLRKHKTNRKPRTPFTTAQLLALERKFRQKQYLSIAERAEFSSSLSLTETQVKIWFQNRRA
Msx3 (Mum) P-PCTLRKHKTNRKPRTPFTTAQLLALERKFHQKQYLSIAERAEFSSSLSLTETQVKIWFQNRRA
Msx3 (Ran) P-PCTLRKHKTNRKPRTPFTTAQLLALERKFHQKQYLSIAERAEFSSSLSLTETQVKIWFQNRRA
Msx3 (Mod) P-PCTLRKHKNNRKPRTPFTTAQLLALERKFRQKQYLSIAERAEFSSSLSLTETQVKIWFQNRRA
MsxB (Dar) T-PCTLRKHKTNRKPRTPFSTSQLLSLERKFRQKQYLSIAERAEFSNSLNLTETQVKIWFQNRRA
MsxB (Fur) A-ACNLRKHKNNRKPRTPFTTSQLLSLERKFRQKQYLSIAERAEFSNSLSLTETQVKIWFQNRRA
MsxC (Dar) P-PCTLRKHKNNRKPRTPFTTSQLLALERKFRQKQYLSIAERAEFSNSLNLTETQVKIWFQNRRA
MsxC (Fur) S-QCNLRKHKNNRKPRTPFTTSQLLALERKFRQKQYLSIAERAEFSNSLNLTETQVKIWFQNRRA
MsxC (Ten) S-QCNLRKHKNNRKPRTPFTTSQLLALERKFRQKQYLSIAERAEFSNSLNLTETQVKIWFQNRRA
Msx (Brf) NK-CTLRKHKTNRKPRTPFTTQQLLALERKFRQKQYLSIAERAEFSASLNLTETQVKIWFQNRRA
Msx (Sak) PKVCTLRKHKTNRKPRTPFTTSQLLALERKFRQKQYLSIAERAEFSASLNLTETQVKIWFQNRRA
Msx (Nev) SK-CQLRKHKANRKPRTPFTTSQLLALERKFRQKQYLSIAERAEFSASLNLTETQVKIWFQNRRA
Msx (Acm) TK-CQLRKHKTNRKPRTPFTTSQLLSLERKFRQKQYLSIAERAEFSASLNLTETQVKIWFQNRRA

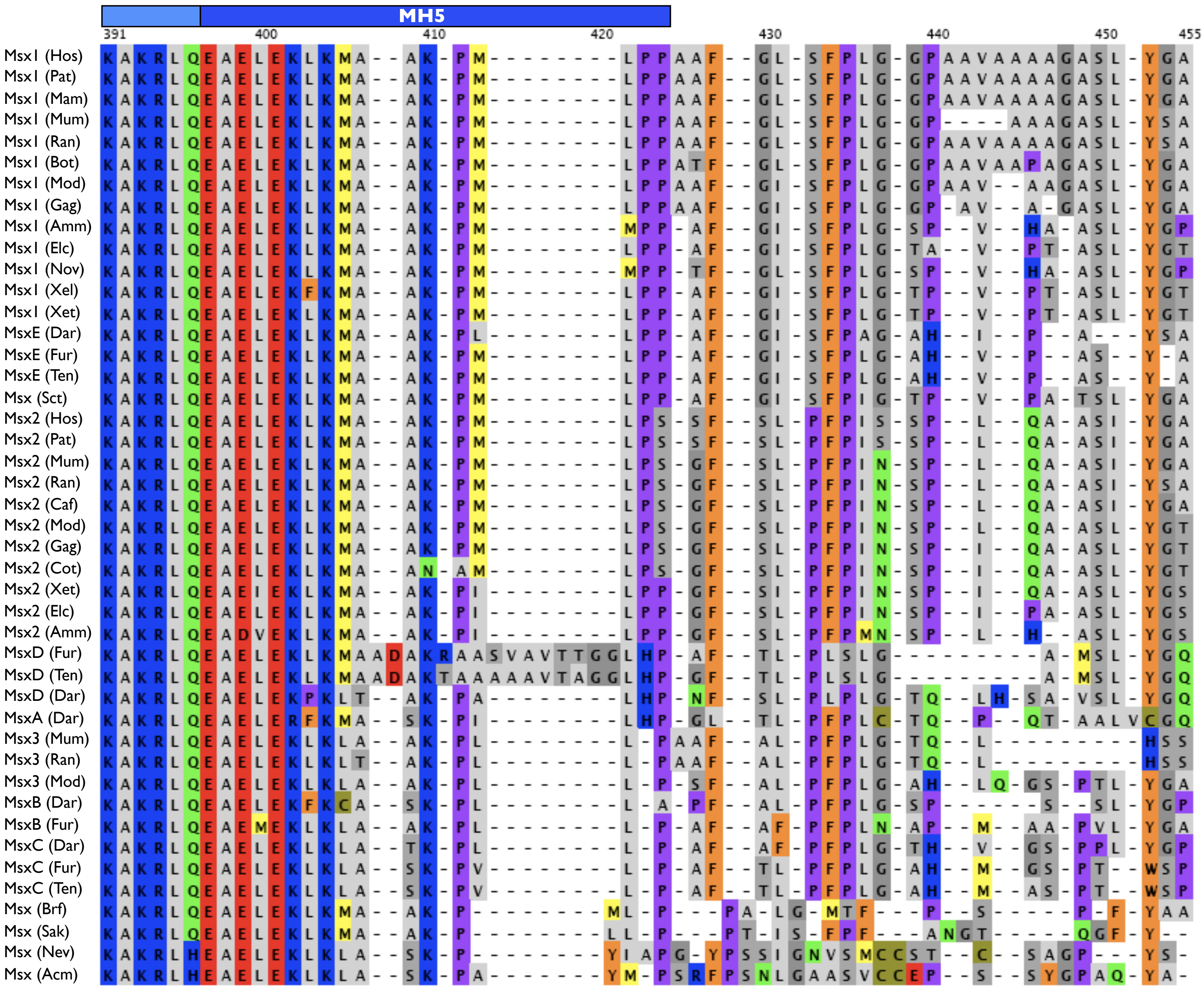

MH5
391
400
410
420
430
440
450
455
Msx1 (Hos)
Msx1 (Pat)
Msx1 (Mam)
Msx1 (Mum)
Msx1 (Ran)
Msx1 (Bot)
Msx1 (Mod)
Msx1 (Gag)
Msx1 (Amm)
Msx1 (Elc)
Msx1 (Nov)
Msx1 (Xel)
Msx1 (Xet)
MsxE (Dar)
MsxE (Fur)
MsxE (Ten)
Msx (Sct)
Msx2 (Hos)
Msx2 (Pat)
Msx2 (Mum)
Msx2 (Ran)
Msx2 (Caf)
Msx2 (Mod)
Msx2 (Gag)
Msx2 (Cot)
Msx2 (Xet)
Msx2 (Elc)
Msx2 (Amm)
MsxD (Fur)
MsxD (Ten)
MsxD (Dar)
MsxA (Dar)
Msx3 (Mum)
Msx3 (Ran)
Msx3 (Mod)
MsxB (Dar)
MsxB (Fur)
MsxC (Dar)
MsxC (Fur)
MsxC (Ten)
Msx (Brf)
Msx (Sak)
Msx (Nev)
Msx (Acm)

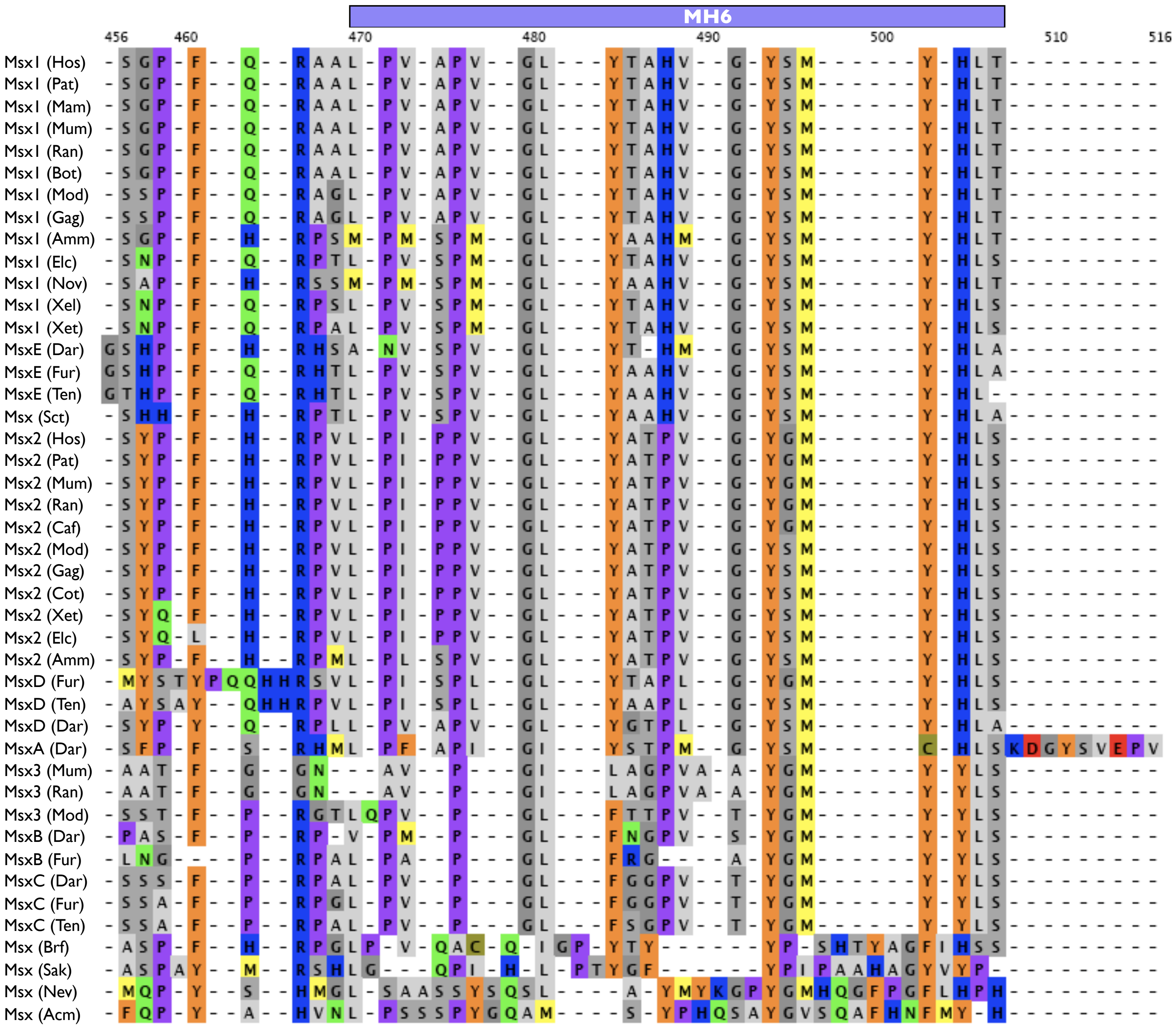
MH6
456
460
470
480
490
500
510
516
MsxI (Hos)
MsxI (Pat)
MsxI (Mam)
MsxI (Mum)
MsxI (Ran)
MsxI (Bot)
MsxI (Mod)
MsxI (Gag)
MsxI (Amm)
MsxI (Elc)
MsxI (Nov)
MsxI (Xel)
MsxI (Xet)
MsxE (Dar)
MsxE (Fur)
MsxE (Ten)
Msx (Sct)
Msx2 (Hos)
Msx2 (Pat)
Msx2 (Mum)
Msx2 (Ran)
Msx2 (Caf)
Msx2 (Mod)
Msx2 (Gag)
Msx2 (Cot)
Msx2 (Xet)
Msx2 (Elc)
Msx2 (Amm)
MsxD (Fur)
MsxD (Ten)
MsxD (Dar)
MsxA (Dar)
Msx3 (Mum)
Msx3 (Ran)
Msx3 (Mod)
MsxB (Dar)
MsxB (Fur)
MsxC (Dar)
MsxC (Fur)
MsxC (Ten)
Msx (Brf)
Msx (Sak)
Msx (Nev)
Msx (Acm)
