# Supplementary material for: Domain duplication, divergence, and loss events in vertebrate Msx paralogs reveal phylogenomically informed disease markers
Source: BMC Evol Biol. 2009 Jan 20;9:18. doi: 10.1186/1471-2148-9-18 (PMC2655272; doi:10.1186/1471-2148-9-18)
Supplement: Additional file 2 — Hidden Markov Model MetaMEME output. This file displays a sample of MetaMEME scores and alignments evaluated against a Hidden Markov Model trained on diverse Msx protein sequences. [file 1471-2148-9-18-S2.doc]

**Additional File 2. Hidden Markov Model MetaMEME output.**

**MEME TRAINING SEQUENCE SET USED FOR METAMEME**

>HsMSX1

MTSLPLGVKVEDSAFGKPAGGGAGQAPSAAAATAAAMGADEEGAKPKVSPSLLPFSVEALMADHRKPGAKESALAPSEGVQAAGGSAQPLGVPPGSLGAPDAPSSPRPLGHFSVGGLLKLPEDALVKAESPEKPERTPWMQSPRFSPPPARRLSPPACTLRKHKTNRKPRTPFTTAQLLALERKFRQKQYLSIAERAEFSSSLSLTETQVKIWFQNRRAKAKRLQEAELEKLKMAAKPMLPPAAFGLSFPLGGPAAVAAAAGASLYGASGPFQRAALPVAPVGLYTAHVGYSMYHLT

>BfMsx_CAA10201

MAQSTLPTSSNSAFSKPTTSAPSSASSPTSTEKLPFSVASLMADKPKETEQNQSDSGPPPLQSPGGPQSPASPPATVPTAPAQPPSRPSDFSVEGILSKPCSSETAAAEKGHDPTGFAAARFPWLQSSRYSPPPRDRLPTPNKCTLRKHKTNRKPRTPFTTQQLLALERKFRQKQYLSIAERAEFSASLNLTETQVKIWFQNRRAKAKRLQEAELEKLKMAAKPMLPPALGMTFPSPFYAAASPFHRPGLPVQACQIGPYTYYPSHTYAGFIHSS

>SkMsx_ABD97280

MSQKSLSLSSNSAFSKPEALPTAQTLSLSTENAENHSSNSTESTTGTMTATAATTAATMTSASIATGTENITFSVASLISHAGHENSKLDDQCRETRHHPHSPSSSTIPSSFSVEGILSKPVSSRDTNSKEEIPLTPTTKSSSWTTATFPWLDSSFSSSNGRSSPPKVCTLRKHKTNRKPRTPFTTSQLLALERKFRQKQYLSIAERAEFSASLNLTETQVKIWFQNRRAKAKRLQEAELEKLKMAAKPLLPPTISFPFANGTQGFYASPAYMRSHLGQPIHLPTYGFYPIPAAHAGYVYP

>HtMsx_AAY86178

MSNKTLTVSNNSAFTRPHHNLSPVTAISPGATPAETPMHKADDRPIKGINFRVESFFSKSPSPLGSSHPSKQHPTDMDCSPVASATPPPENPAARTNSSSSSSFASHSVENILAKSSSSSSSSSEERGRCTSPTTQTYTWNASSFPWMQATRLSPSSGSSDVRPPFAGPKVQCTLRKHKTNRKPRTPFTTSQLLALERKFRQKQYLSIAERAEFSASLNLTETQVKIWFQNRRAKAKRLQEAELEKLKMAAKPMFAPGLGMHIPAAAASAYYSSLGPLHAALRPQISISPYHLGPYAYLPSPPAPSNIVYPYTSGSF

>PdMsx_CAJ38810

MSVLTESRTMHPSSLPALIPSTLHPSSNSAFKPPSQSDRDLKDRITERMPERERMPASPTYSHTSRSIASDSDRDSPLSVTSCSPTRDMLRDGRRSPECRNYSTKRTPSPPHTVTTHKPLSFSVDSIISSTRHRSPSRGESSPRNSPSPVTGPRAPHSPGSSSSSAGVALPPGHSPHGAFSVDGILSKPPHHLATPGALKEGYPTPPYMTSPEAARWAQVAVSAGPFPWLGAPRLSTSPPRPGSPIRKPPTTVQLRKHKTNRKPRTPFTTQQLLALERKFRQKQYLSIAERAEFSASLNLTETQVKIWFQNRRAKAKRLQEAELEKLKMAAKPLLPPGLNMYPGAAAAAAAFYGAQLQRGLPMQPIFHPQYSMFSGHGAPPPSLLSFPH

>Acm_Msx3_ABK41269

MEQSRPAIARRISFSVESLISKPDQNGDITANSTRSSGLPINFSVERLLDKQDVRGGEAAKSSEVNVDVPSERKDINERVDWAEDFPWIHSTRYDPPPRIRPRLSPTKCQLRKHKTNRKPRTPFTTSQLLSLERKFRQKQYLSIAERAEFSASLNLTETQVKIWFQNRRAKAKRLHEAELEKLKLASKPAYMPSRFPSNLGAASVCCEPSSYGPAQYAFQPYAHVNLPSSSPYGQAMSYPHQSAYGVSQAFHNFMYH

**LOCAL DATABASE SEARCH RESULTS**

| ID | Score | Hits | Span | Start | End | Length | Description |
| --- | --- | --- | --- | --- | --- | --- | --- |
| [HsMSX1](http://www.ncbi.nlm.nih.gov/entrez/query.fcgi?db=Protein&cmd=Search&term=HsMsx1&doptcmdl=GenBank) | 399.98 | 5 | 269 | 13 | 281 | 297 | NP 002439.2 Homo sapiens |
| [StMsx](http://www.ncbi.nlm.nih.gov/entrez/query.fcgi?db=Protein&cmd=Search&term=StMsx&doptcmdl=GenBank) | 381.96 | 5 | 214 | 53 | 266 | 282 | BAE98267 Scyliorhinus torazame |
| [AcmMsx3](http://www.ncbi.nlm.nih.gov/entrez/query.fcgi?db=Protein&cmd=Search&term=AcmMsx3&doptcmdl=GenBank) | 363.22 | 5 | 233 | 12 | 244 | 257 | ABK41269 Acropora millepora |
| [NevMsx1](http://www.ncbi.nlm.nih.gov/entrez/query.fcgi?db=Protein&cmd=Search&term=NevMsx1&doptcmdl=GenBank) | 352.87 | 5 | 261 | 8 | 268 | 268 | BAG11598 Nematostella vectensis |
| [AmqMsx](http://www.ncbi.nlm.nih.gov/entrez/query.fcgi?db=Protein&cmd=Search&term=AmqMsx&doptcmdl=GenBank) | 183.26 | 4 | 376 | 3 | 378 | 380 | Msx Amphimedon queenslandica |
| [EflMsx](http://www.ncbi.nlm.nih.gov/entrez/query.fcgi?db=Protein&cmd=Search&term=EflMsx&doptcmdl=GenBank)Prox3 | 165.00 | 1 | 68 | 9 | 76 | 157 | AAA20151 Ephydatia fluviatilis |
| [AmqBshL](http://www.ncbi.nlm.nih.gov/entrez/query.fcgi?db=Protein&cmd=Search&term=AmqBshL&doptcmdl=GenBank) | 128.31 | 3 | 319 | 11 | 329 | 403 | ACA04743 Amphimedon queenslandica |
| [NevNK1](http://www.ncbi.nlm.nih.gov/entrez/query.fcgi?db=Protein&cmd=Search&term=NevNK1&doptcmdl=GenBank) | 99.77 | 3 | 167 | 50 | 216 | 275 | NK1 Nematostella vectensis |
| [PdTlx](http://www.ncbi.nlm.nih.gov/entrez/query.fcgi?db=Protein&cmd=Search&term=PdTlx&doptcmdl=GenBank) | 87.51 | 4 | 235 | 15 | 249 | 325 | ABQ10643 Platynereis dumerilii |
| [PdNK1](http://www.ncbi.nlm.nih.gov/entrez/query.fcgi?db=Protein&cmd=Search&term=PdNK1&doptcmdl=GenBank) | 81.81 | 1 | 68 | 195 | 262 | 333 | CAJ38797 Platynereis dumerilii |
| [AmqNK2-3-4L](http://www.ncbi.nlm.nih.gov/entrez/query.fcgi?db=Protein&cmd=Search&term=AmqNK2-3-4L&doptcmdl=GenBank) | 77.79 | 2 | 276 | 83 | 358 | 359 | ACA04745 Amphimedon queenslandica |
| [SbNK2-3-4L](http://www.ncbi.nlm.nih.gov/entrez/query.fcgi?db=Protein&cmd=Search&term=SbNK2-3-4L&doptcmdl=GenBank) | 76.49 | 5 | 341 | 5 | 345 | 345 | CAD37942 Suberites domuncula |
| [AmqBarH](http://www.ncbi.nlm.nih.gov/entrez/query.fcgi?db=Protein&cmd=Search&term=AmqBarH&doptcmdl=GenBank) | 69.26 | 4 | 270 | 9 | 278 | 313 | BarH Amphimedon queenslandica |
| [AmqTlxLProx2](http://www.ncbi.nlm.nih.gov/entrez/query.fcgi?db=Protein&cmd=Search&term=AmqTlxLProx2&doptcmdl=GenBank) | 64.75 | 2 | 226 | 86 | 311 | 311 | ACA04744 Amphimedon queenslandica |
| [EflNK2L](http://www.ncbi.nlm.nih.gov/entrez/query.fcgi?db=Protein&cmd=Search&term=EflNK2L&doptcmdl=GenBank) | 62.73 | 1 | 68 | 24 | 91 | 118 | AAA20149 Ephydatia fluviatilis |
| [PsDemox](http://www.ncbi.nlm.nih.gov/entrez/query.fcgi?db=Protein&cmd=Search&term=PsDemox&doptcmdl=GenBank) | 59.07 | 1 | 68 | 4 | 71 | 82 | AAX77088 Potamolepis sp. |
| [EmEmH-3](http://www.ncbi.nlm.nih.gov/entrez/query.fcgi?db=Protein&cmd=Search&term=EmEmH-3&doptcmdl=GenBank) | 59.07 | 1 | 68 | 87 | 154 | 166 | AAC18965 Ephydatia muelleri |
| [BiDemox](http://www.ncbi.nlm.nih.gov/entrez/query.fcgi?db=Protein&cmd=Search&term=BiDemox&doptcmdl=GenBank) | 59.07 | 1 | 68 | 4 | 71 | 81 | AAX77090 Baikalospongia intermedia |
| [SdHoxa1](http://www.ncbi.nlm.nih.gov/entrez/query.fcgi?db=Protein&cmd=Search&term=SdHoxa1&doptcmdl=GenBank) | 54.62 | 1 | 68 | 106 | 173 | 364 | CAD37941 Suberites domuncula |
| [EflEmH-3](http://www.ncbi.nlm.nih.gov/entrez/query.fcgi?db=Protein&cmd=Search&term=EflEmH-3&doptcmdl=GenBank) | 54.22 | 1 | 68 | 69 | 136 | 164 | AAB04117 Ephydatia fluviatilis |
| [SlEmH-3](http://www.ncbi.nlm.nih.gov/entrez/query.fcgi?db=Protein&cmd=Search&term=SlEmH-3&doptcmdl=GenBank) | 9.78 | 1 | 29 | 6 | 34 | 137 | AAP75575 Spongilla lacustris |
| [ThEmH-3](http://www.ncbi.nlm.nih.gov/entrez/query.fcgi?db=Protein&cmd=Search&term=ThEmH-3&doptcmdl=GenBank) | 5.58 | 1 | 10 | 19 | 28 | 148 | AAP75576 Trochospongilla horrida |
| [EfrEmH-3](http://www.ncbi.nlm.nih.gov/entrez/query.fcgi?db=Protein&cmd=Search&term=EfrEmH-3&doptcmdl=GenBank) | 5.18 | 1 | 10 | 71 | 80 | 145 | AAP75574 Eunapius fragilis |

**ALIGNMENTS**

| ID Score | Alignment |
| --- | --- |
| [HsMSX1](http://www.ncbi.nlm.nih.gov/entrez/query.fcgi?db=Protein&cmd=Search&term=HsMsx1&doptcmdl=GenBank) 399.98 | 2.9e+01  *______5__*  ........................................**INFSVESLMSH**...............................................  ++FSVE+LM++  1 MTSLPLGVKVEDSAFGKPAGGGAGQAPSAAAATAAAMGADEEGAKPKVSPSL**LPFSVEALMAD**HRKPGAKESALAPSEGVQAAGGSAQPLGVPPGSLGAPDAPSSPRPLG 110  2.5e+01 3.1e+01 2.4e+02  *_____3__* *_____4__* *__________________________________1___________________________  .**FSVEGILSKP**...............**FPWMQSPRYS**...........**CTLRKHKTNRKPRTPFTTQQLLALERKFRQKQYLSIAERAEFSASLNLTETQVKIWFQNRRAK**  FSV+G+L++P +PWMQSPR+S CTLRKHKTNRKPRTPFTT+QLLALERKFRQKQYLSIAERAEFS+SL+LTETQVKIWFQNRRAK  111 H**FSVGGLLKLP**EDALVKAESPEKPER**TPWMQSPRFS**PPPARRLSPPA**CTLRKHKTNRKPRTPFTTAQLLALERKFRQKQYLSIAERAEFSSSLSLTETQVKIWFQNRRAK** 220  6.6e+01  ____**___________2_______*  **AKRLQEAELEKLKMAAKPMLPPGLFM**...................................  AKRLQEAELEKLKMAAKPMLPP++F+  221 **AKRLQEAELEKLKMAAKPMLPPAAFG**LSFPLGGPAAVAAAAGASLYGASGPFQRAALPVAPVGLYTAHVGYSMYHLT |
| [StMsx](http://www.ncbi.nlm.nih.gov/entrez/query.fcgi?db=Protein&cmd=Search&term=StMsx&doptcmdl=GenBank) 381.96 | 2.9e+01 1.3e+01  *______5__* *_____3  **INFSVESLMSH**........................................**FSVEGIL**  ++FSVE+LMS+ + VEG+L  1 MLQLERDRLYMAPCSVMSAPQVTTGGVRSDEPRSLANVKLAAAEEMDRPKAP**LPFSVEALMSD**RKPSRERAASEAALGGTSQSLSPRMAGQETAATPLAATTS**YTVEGLL** 110  2.7e+01 2.5e+02 6.0e  __* *_____4__* *__________________________________1_______________________________**___  **SKP**...............**FPWMQSPRYS**..........**CTLRKHKTNRKPRTPFTTQQLLALERKFRQKQYLSIAERAEFSASLNLTETQVKIWFQNRRAKAKRLQEAEL**  + + +PWMQ PR+S CTLRKHKTNRKPRTPFTT+QLLALERKFRQKQYLSIAERAEFS+SLNLTETQVKIWFQNRRAKAKRLQEAEL  111 **KIS**EEALVKSESGERQER**TPWMQDPRFS**PPPRRMSPPA**CTLRKHKTNRKPRTPFTTSQLLALERKFRQKQYLSIAERAEFSSSLNLTETQVKIWFQNRRAKAKRLQEAEL** 220  +01  ________2_______*  **EKLKMAAKPMLPPGLFM**.............................  EKLKMAAKPMLPP+ +  221 **EKLKMAAKPMLPPAFGI**SFPIGTPVPATSLYGASHHFHRPTLPVSPVGLYAAHVGYSMYHLA |
| [AcmMsx3](http://www.ncbi.nlm.nih.gov/entrez/query.fcgi?db=Protein&cmd=Search&term=AcmMsx3&doptcmdl=GenBank) 363.22 | 2.9e+01 1.6e+01 3.0e+01 2.  *______5__* *_____3__* *_____4__* *_  **INFSVESLMSH**....................**FSVEGILSKP**.................................**FPWMQSPRYS**.............**CT**  I+FSVESL+S+ FSVE +L K FPW++S+RY+ C+  1 MEQSRPAIARR**ISFSVESLISK**PDQNGDITANSTRSSGLPIN**FSVERLLDKQ**DVRGGEAAKSSEVNVDVPSERKDINERVDWAED**FPWIHSTRYD**PPPRIRPRLSPTK**CQ** 110  5e+02 5.6e+01  _________________________________1_______________________________**___________2_______*  **LRKHKTNRKPRTPFTTQQLLALERKFRQKQYLSIAERAEFSASLNLTETQVKIWFQNRRAKAKRLQEAELEKLKMAAKPMLPPGLFM**.......................  LRKHKTNRKPRTPFTT+QLL+LERKFRQKQYLSIAERAEFSASLNLTETQVKIWFQNRRAKAKRL+EAELEKLK+A+KP+++P++F+  111 **LRKHKTNRKPRTPFTTSQLLSLERKFRQKQYLSIAERAEFSASLNLTETQVKIWFQNRRAKAKRLHEAELEKLKLASKPAYMPSRFP**SNLGAASVCCEPSSYGPAQYAFQ 220      ........................    221 PYAHVNLPSSSPYGQAMSYPHQSAYGVSQAFHNFMYH |
| [NevMsx1](http://www.ncbi.nlm.nih.gov/entrez/query.fcgi?db=Protein&cmd=Search&term=NevMsx1&doptcmdl=GenBank) 352.87 | 2.4e+01 2.1e+01 3.2e+01  *______5__* *_____3__* *_____4__*  ...................**INFSVESLMSH**...................**FSVEGILSKP**................................**FPWMQSPRYS**..  +FSVESL+S+ FSVE+IL K FPWM+S+RY+  1 MEADRDLPSPSKAVPTTSTASITSRT**SSFSVESLISK**PEQQPSNKRKPYTPGLQSN**FSVESILEKQ**SSRDEESQQRKVATTTAEAGKKSSEDGSTTED**FPWMHSTRYD**PP 110  2.4e+02 3.8e+01  *__________________________________1_______________________________**___________2_______*  ...........**CTLRKHKTNRKPRTPFTTQQLLALERKFRQKQYLSIAERAEFSASLNLTETQVKIWFQNRRAKAKRLQEAELEKLKMAAKPMLPPGLFM**..........  C+LRKHK NRKPRTPFTT+QLLALERKFRQKQYLSIAERAEFSASLNLTETQVKIWFQNRRAKAKRL+EAELEKLK+A+KP +PG  111 PRIRTRLSPSK**CQLRKHKANRKPRTPFTTSQLLALERKFRQKQYLSIAERAEFSASLNLTETQVKIWFQNRRAKAKRLHEAELEKLKLASKPYIAPGYPS**SIGNVSMCCS 220      ................................................    221 TCSAGPYSMQPYSHMGLSAASSYSQSLAYMYKGPYGMHQGFPGFLHPH |
| [AmqMsx](http://www.ncbi.nlm.nih.gov/entrez/query.fcgi?db=Protein&cmd=Search&term=AmqMsx&doptcmdl=GenBank) 183.26 | ............................................................................................................    1 MDRLPFTQLFQHGYLASTTTKPNAMPLRPVPIQSPTVAEPYPIISTSSSSYHHHHNLQQHPSDNMPYAVQATTSVPAAVRPSSCSAAAAIQSSPPPPYQSELLDPREQSA 110  7.5e+00 2.4e+00 1.6e+02  *_____3__* *_____4__* *____________________________  ............................**FSVEGILSKP**.................**FPWMQSPRYS**................**CTLRKHKTNRKPRTPFTTQQLLALERKFR**  +S LS+P FP SP L K K RKPRTPFT QL ALERKFR  111 AAYLTNSTLAGGVQTASMMPISSHPPSL**HSSPHCLSLP**IMAREPPGSAGLDPNTG**FPKVSSPTIH**TPDGSQSVSGSPTIGQ**AKLKKQKKDRKPRTPFTSTQLIALERKFR** 220  -9.8e+00  ______1_______________________________**___________2_______*  **QKQYLSIAERAEFSASLNLTETQVKIWFQNRRAKAKRLQEAELEKLKMAAKPMLPPGLFM**..................................................  Q YLS AERAEF L LTETQVKIWFQNRRAK KRL EAE E L +  221 **QQRYLSVAERAEFAEYLKLTETQVKIWFQNRRAKEKRLREAEAERAARSLGIPLSYAHYH**TELLHNPLIHGSGMSLQPNAAANTLGLFQQGPLIASPPVPHTPHYPHSQA 330    ................................................    331 LHFNHPHQPHPTSSIKYEGQSPQQQSSPATLGFMLDPNRSSLSVPHTTIS |
| EflMsxProx3 165.00 | 1.7e+02  *__________________________________1_______________________________*  **CTLRKHKTNRKPRTPFTTQQLLALERKFRQKQYLSIAERAEFSASLNLTETQVKIWFQNRRAKAKRLQ**  T K K RKPRTPFT QL ALERKFRQ YLS AERAEF L LTETQVKIWFQNRRAK KRL+  1 PHSSGSNA**STINKQKKDRKPRTPFTSTQLIALERKFRQQKYLSVAERAEFAEYLKLTETQVKIWFQNRRAKEKRLH**EAEAERAARSLGFHFHMPMQSKMNTFRHPYCNSQ 110 |
| [AmqBshL](http://www.ncbi.nlm.nih.gov/entrez/query.fcgi?db=Protein&cmd=Search&term=AmqBshL&doptcmdl=GenBank) 128.31 | ....................................................................................................    1 MKTGLLLHYNQPSASPSSKMMLATYPSPAHQQQQATSPPAFSAISQRTHPAAGGLLSTATGFIPTALPLAIAPSAVFGHHHHHHQPVALRGPVPAPQILQHHFASVKVEE 110  1.7e+01  *_____3__*  .............................**FSVEGILSKP**.......................................................................  FS + ILSK  111 AKRRSESPRSRSPSSTPPAPQASKPQHTA**FSIDAILSKK**DEPKKHISSSSTSSEVSLAPHQTSLLASPPSVGHTPHLTRTTANGLFYIYTAASPTATGGHPTVVQAAQNP 220  5.8e-02 8.3e+01  *_____4__* *__________________________________1_______________________________*  .....**FPWMQSPRYS**..........................**CTLRKHKTNRKPRTPFTTQQLLALERKFRQKQYLSIAERAEFSASLNLTETQVKIWFQNRRAKAKRLQ**  F + ++ K K RK RT FT QL LE KF K YLS R E L L ETQVK WFQNRR K K Q  221 SPFSP**FASLRGHHFD**HHHHHHHELQRSPLGPLVLPPPEPSV**LLSSKLKRKRKLRTVFTEKQLEGLETKFSEKKYLSVPDRMELANRLELSETQVKTWFQNRRMKCKKQQ**Q 330 |
| [NevNK1](http://www.ncbi.nlm.nih.gov/entrez/query.fcgi?db=Protein&cmd=Search&term=NevNK1&doptcmdl=GenBank) 99.77 | 8.7e+00 1.9e+01  *_____3__* *_____4__*  **FSVEGILSKP**.................................**FPWMQSPRYS**........  FSV IL PWM ++RY  1 MLRPDCAPDQSAMEARVNGVIEEPVSDSDSQPDAGTYSTHEELRPVVTS**FSVKDILDPN**KFTASTIRRRSSDSDGDSDAEGSSTPTSPTGVW**HPWMNATRYN**RPQTKSQD 110  8.3e+01  *__________________________________1_______________________________*  ......................................**CTLRKHKTNRKPRTPFTTQQLLALERKFRQKQYLSIAERAEFSASLNLTETQVKIWFQNRRAKAKRLQ**  K R RT FT QL ALE KF YLS ER SL LTETQVKIWFQNRR K K  111 PDKDKAPDVDISRSDQETTDVAGEDPKSSSKSKQKRGS**TERAKEGKPRRARTAFTYEQLVALENKFKSTRYLSVCERLNLALSLGLTETQVKIWFQNRRTKWKKQN**PGMD 220 |
| [PdTlx](http://www.ncbi.nlm.nih.gov/entrez/query.fcgi?db=Protein&cmd=Search&term=PdTlx&doptcmdl=GenBank) 87.51 | 1.2e+01  *______5__*  ..................................................**INFSVESLMSH**...................................  ++F +++S+  1 MDIDENSMESVDIETRSTPSPADRGCGSVSPPVAVDASPGSSRSTPGPSSSPGHQEHSPTNSGK**LSFGISRILSD**DMGASDKPHHDSTPHSAHPRDLLQHSMAYSARIFG 110  -7.9e-01 1.2e+01 6.4e+01  *_____3__* *_____4__* *__________________________________1___  ....................................**FSVEGILSKP**.....**FPWMQSPRYS**..........**CTLRKHKTNRKPRTPFTTQQLLALERKFRQKQYLSIAER**  + V + S FPWMQ + R KPRT FT Q LE F YL AER  111 APYPVVLGRAGSPEAGESPGLIRVPIHRPVPFSPHP**HNVPNPHSPG**FSPLM**FPWMQDRKDR**LTVSRRIGHP**YQNRTPPKRKKPRTSFTRLQIIELEKRFHRQKYLASAER** 220    ____________________________*  **AEFSASLNLTETQVKIWFQNRRAKAKRLQ**  SL T QVK WFQNRR K R  221 **SALAKSLKMTDAQVKTWFQNRRTKWRRQT**AEEREAERQAASRFMLGLQAEAGGSIYKNPDPLCMNNASLHALQRLQPWADEKGNIDGERPAYLNASSLHSPASVL |
| [PdNK1](http://www.ncbi.nlm.nih.gov/entrez/query.fcgi?db=Protein&cmd=Search&term=PdNK1&doptcmdl=GenBank) 81.81 | 8.2e+01  *________________________  **CTLRKHKTNRKPRTPFTTQQLLALE**  R RT FT QL ALE  110 ADILDPGKFTGRDKRRKQNSPMCLAPLGGHHLRADRVEDSQGAYSVSENGENEAREEHTLEHEDAFSMSLDSSKNEGDISDSESD**KDGGRGGKPRRARTAFTYEQLVALE** 219    __________1_______________________________*  **RKFRQKQYLSIAERAEFSASLNLTETQVKIWFQNRRAKAKRLQ**  KF YLS ER SLNLTETQVKIWFQNRR K K  220 **NKFKSTRYLSVCERLNLALSLNLTETQVKIWFQNRRTKWKKQN**PGLDINTPTIPSTPSSSGFGLHHPYSLSSLYGQSLHPYLSSTSGALGLLRSPPGALSGHPQIYYPYF 329 |
| [AmqNK2-3-4L](http://www.ncbi.nlm.nih.gov/entrez/query.fcgi?db=Protein&cmd=Search&term=AmqNK2-3-4L&doptcmdl=GenBank) 77.79 | 6.6e+0  *_____  ......................**CTLRKH**  T  1 MSSGKKKSFMICDILDDIYTSDSSRPSSTKLQVHPHIVHSSSVIYTASAAPSHIEGGLVGGEGDECSPGTDSGLGDCKPEAIRPESPTQHEPDSPSSSQSSEQG**STGAAK** 110  1 -1.2e+01  _____________________________1_______________________________* *___________2_______*  **KTNRKPRTPFTTQQLLALERKFRQKQYLSIAERAEFSASLNLTETQVKIWFQNRRAKAKRLQ**..**EAELEKLKMAAKPMLPPGLFM**.........................  R PR F +Q LER + YL ER L LTETQVKIWFQNRR K KR Q A L A LF+  111 **TRKRRPRGLFSHAQIYELERRYALQKYLTAHEREQLANMLRLTETQVKIWFQNRRYKNKRQQ**LE**NARLSPKSAVAACSKTSDLFP**SAIPPPPPLHSIAAAPSDIKLPSTT 220      ..............................................................................................................    221 PAFPVNSPLSLNLTHAPPSALPIGVVATSLITPSGTQSPLYSITGPPPSLPPPSDYSFRYPSGHPPPIMNIKSTPPSLPKSMYYPAVAAAYGGSVTSVNATIPSGSYVSS 330      ............................    331 ICSCTTMPYQPLPRVPSPATSVTSIRSQY |
| [SbNK2-3-4L](http://www.ncbi.nlm.nih.gov/entrez/query.fcgi?db=Protein&cmd=Search&term=SbNK2-3-4L&doptcmdl=GenBank) 76.49 | 2.5e+00 2.6e+00 4.5e  *______5__* *_____3__* *___  **INFSVESLMSH**....................................................**FSVEGILSKP**.............................**FPWM**  I+ S M+ F + IL +  1 MTRQ**IPNQPQSAMAN**ECRHQCFHGSSTTHRESTNGSQTTKERVVFRAPSVPAATHGHHLPTSTHRTS**FMIDDILQPT**PNTRSSRTISSSSGLTEDGSYCSTFNEES**TRRS** 110  +00 7.1e+01 -1.7e+01  __4__* *__________________________________1_______________________________**_______  **QSPRYS**............................**CTLRKHKTNRKPRTPFTTQQLLALERKFRQKQYLSIAERAEFSASLNLTETQVKIWFQNRRAKAKRLQEAELEKLK**  +SPR+ R PR F +Q LER F YL ER + L LTETQVKIWFQNRR K KR Q  111 **DSPRSL**FSEDAGREGSPEGSDSDTERPSSQGSNS**SSSPSKTKKRRPRALFSHAQVFELERRFAVQKYLTAHEREQLASMLHLTETQVKIWFQNRRYKNKRQQIEQQRLSP** 220    ____2_______*  **MAAKPMLPPGLFM**.................................................................................................  A K M L +  221 **KACKDMTKSLLHP**SVKSPPTTFPIATLGPVHLGLSSPQPRSVLPTSQHQPVYSISGSEYFRYPSVPAALMRPSVTALPNSLYYPHTSVSSTLRPFAPLTPATFSPYHPLP 33    ...............    331 QALKVPAAGDSYAHA |
| [AmqBarH](http://www.ncbi.nlm.nih.gov/entrez/query.fcgi?db=Protein&cmd=Search&term=AmqBarH&doptcmdl=GenBank) 69.26 | 1.7e+01 -1.1e+00 9  *______5__* *_____3__* *  **INFSVESLMSH**.......................................................................**FSVEGILSKP**.........**F**  ++FSV++L+ F V + + +  1 MEVKTTRS**LSFSVDRLLQP**SPAEQAAKKMSDQSSSGGSSTSSSPPSSPFSSPIVGGATKAATPAHPIFLAAARTGTPPRHNIMTLNTTFP**FPVTLPFAHS**FSPFALCAA**T** 110  .1e+00 4.9e+01  _____4__* *_________  **PWMQSPRYS**...........................................................................................**CTLRKHKTNR**  W Q ++ S K  111 **NWGQPSFTS**WSLGQPGGAPGLPAKLAPPSATGWSLHPAQLHKPQHITCSTTKRILELPKVGTPIVAVPDDLEDYTGGDDDSDSSSPGPSSSEHGGADDGE**PGKIGKKYKK** 220    _________________________1_______________________________*  **KPRTPFTTQQLLALERKFRQKQYLSIAERAEFSASLNLTETQVKIWFQNRRAKAKRLQ**  K RT FT +QL LE F Q YL R SL LTE K W+QNRR K KR  221 **KKRTTFTSSQLQQLETRFNQQKYLTKLDRCRMAQSLGLTEKHIKTWYQNRRTKWKREC**TDEMWSRERETAAANMYTQHLQLKSINGSSPLTIS |
| [AmqTlxLProx2](http://www.ncbi.nlm.nih.gov/entrez/query.fcgi?db=Protein&cmd=Search&term=AmqTlxLProx2&doptcmdl=GenBank) 64.75 | 6.2e+01  *________________________  **CTLRKHKTNRKPRTPFTTQQLLALE**  KPRT F Q LE  1 MESCCTALNQIQPQLQNSFRIDSILTGMGAHPPPPPQGYSVMHQHPSNAGGPGGVEAIKQFGNMFGKNEELNMQERKRESVSSAD**DDDDSYRKKKKPRTAFSREQVSELE** 110  -3.5e+00  __________1_______________________________* *___________2_______*  **RKFRQKQYLSIAERAEFSASLNLTETQVKIWFQNRRAKAKRLQ**...**EAELEKLKMAAKPMLPPGLFM**...........................................  KF YLS AER E L L QVK WFQNRR K KR E E K P P + F  111 **KKFTERKYLSSAERGELAEKLKLSDMQVKTWFQNRRMKFKRQN**EEA**ELEVKSPKFPYPPFVPYSSFY**SYTMPHYKMMDQMQQQQQQQQQSPSYTCTSPPSTSNSYQQSTP 220      ...........................................................................................    221 GPTPLVLGTNPAAHHISPPPHITSTYFNRAAIAHGVISNGSQSPCYHIGAGYLSDYNPGAPSGPIMSPPYCNSSGSSDWQRSPLPTPPAAP |
| [EflNK2L](http://www.ncbi.nlm.nih.gov/entrez/query.fcgi?db=Protein&cmd=Search&term=EflNK2L&doptcmdl=GenBank) 62.73 | 6.3e+01  *__________________________________1_______________________________*  **CTLRKHKTNRKPRTPFTTQQLLALERKFRQKQYLSIAERAEFSASLNLTETQVKIWFQNRRAKAKRLQ**  R PR F +Q LER F YL E L LTETQVKIWFQNRR K KR Q  1 NSDEDKDRYASDLDTDRASSAGG**ALQMSRHKKRRPRALFSHAQVYELERRFAVQKYLTAHEQSKLATVLHLTETQVKIWFQNRRYKSKRQQ**IEQTRVSPKVVKTSRMVRC 110 |
| [PsDemox](http://www.ncbi.nlm.nih.gov/entrez/query.fcgi?db=Protein&cmd=Search&term=PsDemox&doptcmdl=GenBank) 59.07 | 5.9e+01  *__________________________________1_______________________________*  **CTLRKHKTNRKPRTPFTTQQLLALERKFRQKQYLSIAERAEFSASLNLTETQVKIWFQNRRAKAKRLQ**  K RT F Q LE KF K YLS AER E L L QVK WFQNRR K KR  1 GND**AEDDLFTRRKKARTAFSREQVAELEKKFQDKKYLSSAERGELAEKLKLSDMQVKTWFQNRRMKYKRQS**EETEMEMKSPK |
| [EmEmH-3](http://www.ncbi.nlm.nih.gov/entrez/query.fcgi?db=Protein&cmd=Search&term=EmEmH-3&doptcmdl=GenBank) 59.07 | 5.9e+01  *_______________________  **CTLRKHKTNRKPRTPFTTQQLLAL**  K RT F Q L  1 MDNCRGDKKPLLSTNQQSFRIDNLLTRKVIEQQQQPDHYTMYPPSKVENHDILSLTTGPSHDDMISDGTEIYEQGRESTSSTSGND**AEDDLLTRRKKARTAFSREQVAEL** 110    ___________1_______________________________*  **ERKFRQKQYLSIAERAEFSASLNLTETQVKIWFQNRRAKAKRLQ**  E KF K YLS AER E L L QVK WFQNRR K KR  111 **EKKFQDKKYLSSAERGELAEKLKLSDMQVKTWFQNRRMKYKRQS**EETEMEMKSPKY |
| [BiDemox](http://www.ncbi.nlm.nih.gov/entrez/query.fcgi?db=Protein&cmd=Search&term=BiDemox&doptcmdl=GenBank) 59.07 | 5.9e+01  *__________________________________1_______________________________*  **CTLRKHKTNRKPRTPFTTQQLLALERKFRQKQYLSIAERAEFSASLNLTETQVKIWFQNRRAKAKRLQ**  K RT F Q LE KF K YLS AER E L L QVK WFQNRR K KR  1 GND**AEDDLLTRRKKARTAFSREQVAELEKKFQDKKYLSSAERGELAEKLKLSDMQVKTWFQNRRMKYKRQS**EETEMEMKSP |
| [SdHoxa1](http://www.ncbi.nlm.nih.gov/entrez/query.fcgi?db=Protein&cmd=Search&term=SdHoxa1&doptcmdl=GenBank) 54.62 | 5.5e+  *____  **CTLRK**    1 MTLNSLWHWIPINLEKRKTQSLAPYQQSFRIDDLLRQKAIEQQPDHFPLPSPDDRFEENGTFRIGPVPPPKSTKTNVEKKISVDSTSPTGSVRNGTKRSISSDIE**DDDEL** 110  01  ______________________________1_______________________________*  **HKTNRKPRTPFTTQQLLALERKFRQKQYLSIAERAEFSASLNLTETQVKIWFQNRRAKAKRLQ**  K RT F Q LE KF K YLS ER E L L QVK WFQNRR K KR  111 **FRKRKKARTAFSREQVAELEKKFQEKKYLSSNERGELAEKLKLSDMQVKTWFQNRRMKFKRQS**EEAEMEMKASKYSFSSFMPYGNMTSLYGYMQNGYPYPTNIRSPRTPS 220 |
| [EflEmH-3](http://www.ncbi.nlm.nih.gov/entrez/query.fcgi?db=Protein&cmd=Search&term=EflEmH-3&doptcmdl=GenBank) 54.22 | 5.4e+01  *__________________________________1______  **CTLRKHKTNRKPRTPFTTQQLLALERKFRQKQYLSIAERAEF**  K RT F Q LE KF K YLS ER E  1 MDNCRGEKKPLLSTNQQSFRIDNLLTRKVIEQQQQPDHYTIYSPSKISDGTKIYEHGRESTSSTSGND**ADDDLLTRRKKARTAFSREQVAELEKKFQNKKYLSSTERGEL** 110    _________________________*  **SASLNLTETQVKIWFQNRRAKAKRLQ**  L L QVK WFQNRR K KR  111 **AEKLKLSDMQVKTWFQNRRMKYKRQS**EETEMEMKSSKYGSFVYRGVAHSMFNAV |
| [SlEmH-3](http://www.ncbi.nlm.nih.gov/entrez/query.fcgi?db=Protein&cmd=Search&term=SlEmH-3&doptcmdl=GenBank) 9.78 | 8.5e+00  *______5__*  **INFSVESLMSH**..................  +F+ + L+ H  1 WSTYQ**QSFRIDDLLTH**KAIEQQQQPDHYSMYPSTKIESNNGILNLTTRTHDDMISDGTEMCEHGRESTSSTSGNDADDDLLTRRKKARTAFSREQVADLEKKFQDKKYLS 110 |
| [ThEmH-3](http://www.ncbi.nlm.nih.gov/entrez/query.fcgi?db=Protein&cmd=Search&term=ThEmH-3&doptcmdl=GenBank) 5.58 | 5.6e+00  *_____3__*  **FSVEGILSKP**  F +++L  1 MDNCRGEKKPPPAANQQS**FRIDNLLTRK**VIEQQQQPDHYTIYSPTKVESHEVLNLTTSPSHDDMISDGTDLYEHGRESTSSLSGNDADDDLLTRRKKARTAFSREQVAEL 110 |
| [EfrEmH-3](http://www.ncbi.nlm.nih.gov/entrez/query.fcgi?db=Protein&cmd=Search&term=EfrEmH-3&doptcmdl=GenBank) 5.18 | 5.2e+00  *_____3__*  **FSVEGILSKP**  +S E+ S +  1 NCRGDKKPPMSTYQQSFRIDDLLTRKVIEQQQQPDHFAIYAPIKMENNDILNLTTIPHEDIISDGTEICE**HSRESTSSTS**GNDAEDDLLTRRKKARTAFSREQVAELEKK 110 |
