# Supplementary figures and images for: Domain duplication, divergence, and loss events in vertebrate Msx paralogs reveal phylogenomically informed disease markers
Source: BMC Evol Biol. 2009 Jan 20;9:18. doi: 10.1186/1471-2148-9-18 (PMC2655272; doi:10.1186/1471-2148-9-18)

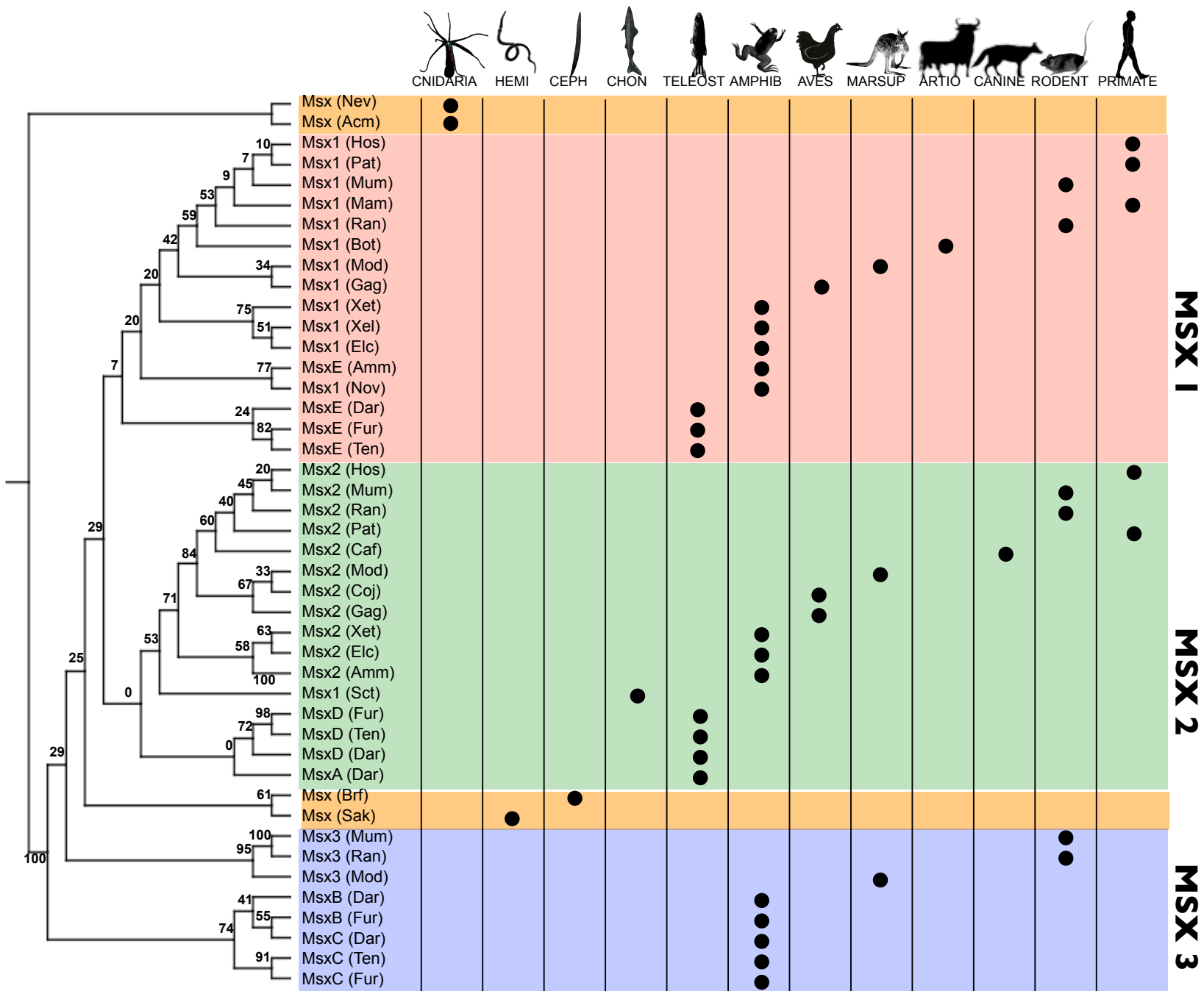

Supplement: Additional file 3 — Msx Phylogeny (without gaps). This file represents the phylogenetic analysis of the ungapped alignment, as described in the text. [file 1471-2148-9-18-S3.pdf]
