# Supplementary material for: Domain duplication, divergence, and loss events in vertebrate Msx paralogs reveal phylogenomically informed disease markers
Source: BMC Evol Biol. 2009 Jan 20;9:18. doi: 10.1186/1471-2148-9-18 (PMC2655272; doi:10.1186/1471-2148-9-18)
Supplement: Additional file 4 — Msx Alignment (without gaps). This file represents the multisequence alignment of Msx protein sequences after all gaps were removed, as described in the text. [file 1471-2148-9-18-S4.pdf]

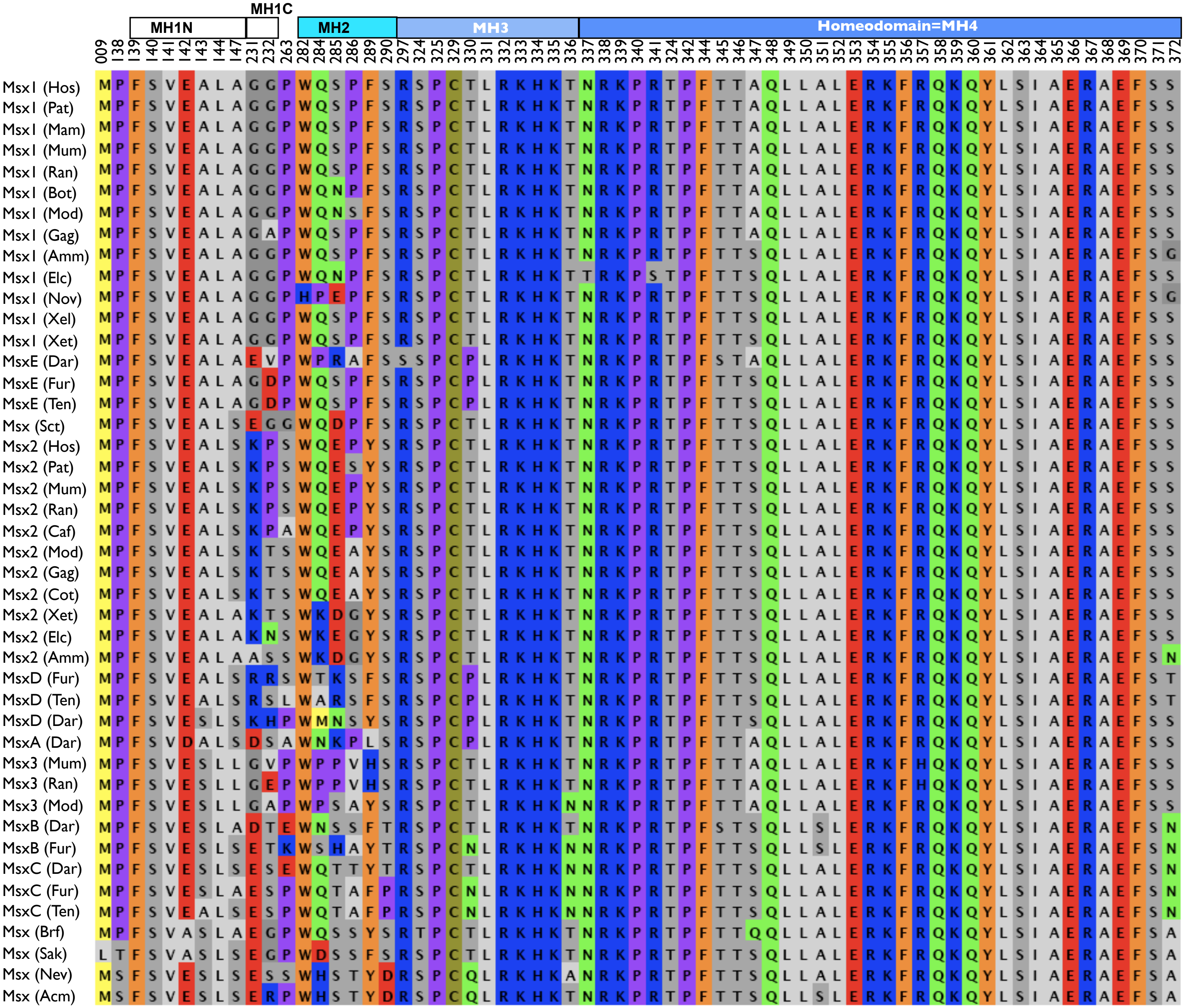
MH1N
MH1C
MH2
MH3
Homeodomain=MH4
009 138 139 140 141 142 143 144 147 231 232 263 282 284 285 286 289 290 297 324 325 329 330 331 332 333 334 335 336 337 338 339 340 341 324 342 344 345 346 347 348 349 350 351 352 353 354 355 356 357 358 359 360 361 362 363 364 365 366 367 368 369 370 371 372
Msx1 (Hos) M P F S V E A L A G G P W Q S P F S R S P C T L R K H K T N R K P R T P F T T A Q L L A L E R K F R Q K Q Y L S I A E R A E F S S
Msx1 (Pat) M P F S V E A L A G G P W Q S P F S R S P C T L R K H K T N R K P R T P F T T A Q L L A L E R K F R Q K Q Y L S I A E R A E F S S
Msx1 (Mam) M P F S V E A L A G G P W Q S P F S R S P C T L R K H K T N R K P R T P F T T A Q L L A L E R K F R Q K Q Y L S I A E R A E F S S
Msx1 (Mum) M P F S V E A L A G G P W Q S P F S R S P C T L R K H K T N R K P R T P F T T A Q L L A L E R K F R Q K Q Y L S I A E R A E F S S
Msx1 (Ran) M P F S V E A L A G G P W Q S P F S R S P C T L R K H K T N R K P R T P F T T A Q L L A L E R K F R Q K Q Y L S I A E R A E F S S
Msx1 (Bot) M P F S V E A L A G G P W Q N P F S R S P C T L R K H K T N R K P R T P F T T A Q L L A L E R K F R Q K Q Y L S I A E R A E F S S
Msx1 (Mod) M P F S V E A L A G G P W Q N S F S R S P C T L R K H K T N R K P R T P F T T A Q L L A L E R K F R Q K Q Y L S I A E R A E F S S
Msx1 (Gag) M P F S V E A L A G A P W Q S P F S R S P C T L R K H K T N R K P R T P F T T A Q L L A L E R K F R Q K Q Y L S I A E R A E F S S
Msx1 (Amm) M P F S V E A L A G G P W Q S P F S R S P C T L R K H K T N R K P R T P F T T S Q L L A L E R K F R Q K Q Y L S I A E R A E F S G
Msx1 (Elc) M P F S V E A L A G G P W Q N P F S R S P C T L R K H K T T R K P S T P F T T S Q L L A L E R K F R Q K Q Y L S I A E R A E F S S
Msx1 (Nov) M P F S V E A L A G G P H P E P F S R S P C T L R K H K T N R K P R T P F T T S Q L L A L E R K F R Q K Q Y L S I A E R A E F S G
Msx1 (Xel) M P F S V E A L A G G P W Q S P F S R S P C T L R K H K T N R K P R T P F T T S Q L L A L E R K F R Q K Q Y L S I A E R A E F S S
Msx1 (Xet) M P F S V E A L A G G P W Q S P F S R S P C T L R K H K T N R K P R T P F T T S Q L L A L E R K F R Q K Q Y L S I A E R A E F S S
MsxE (Dar) M P F S V E A L A E V P W P R A F S S S P C P L R K H K T N R K P R T P F S T A Q L L A L E R K F R Q K Q Y L S I A E R A E F S S
MsxE (Fur) M P F S V E A L A G D P W Q S P F S R S P C P L R K H K T N R K P R T P F T T S Q L L A L E R K F R Q K Q Y L S I A E R A E F S S
MsxE (Ten) M P F S V E A L A G D P W Q S P F S R S P C P L R K H K T N R K P R T P F T T S Q L L A L E R K F R Q K Q Y L S I A E R A E F S S
Msx (Sct) M P F S V E A L S E G G W Q D P F S R S P C T L R K H K T N R K P R T P F T T S Q L L A L E R K F R Q K Q Y L S I A E R A E F S S
Msx2 (Hos) M P F S V E A L S K P S W Q E P Y S R S P C T L R K H K T N R K P R T P F T T S Q L L A L E R K F R Q K Q Y L S I A E R A E F S S
Msx2 (Pat) M P F S V E A L S K P S W Q E S Y S R S P C T L R K H K T N R K P R T P F T T S Q L L A L E R K F R Q K Q Y L S I A E R A E F S S
Msx2 (Mum) M P F S V E A L S K P S W Q E P Y S R S P C T L R K H K T N R K P R T P F T T S Q L L A L E R K F R Q K Q Y L S I A E R A E F S S
Msx2 (Ran) M P F S V E A L S K P S W Q E P Y S R S P C T L R K H K T N R K P R T P F T T S Q L L A L E R K F R Q K Q Y L S I A E R A E F S S
Msx2 (Caf) M P F S V E A L S K P A W Q E P Y S R S P C T L R K H K T N R K P R T P F T T S Q L L A L E R K F R Q K Q Y L S I A E R A E F S S
Msx2 (Mod) M P F S V E A L S K T S W Q E A Y S R S P C T L R K H K T N R K P R T P F T T S Q L L A L E R K F R Q K Q Y L S I A E R A E F S S
Msx2 (Gag) M P F S V E A L S K T S W Q E A Y S R S P C T L R K H K T N R K P R T P F T T S Q L L A L E R K F R Q K Q Y L S I A E R A E F S S
Msx2 (Cot) M P F S V E A L S K T S W Q E A Y S R S P C T L R K H K T N R K P R T P F T T S Q L L A L E R K F R Q K Q Y L S I A E R A E F S S
Msx2 (Xet) M P F S V E A L A K T S W K D G Y S R S P C T L R K H K T N R K P R T P F T T S Q L L A L E R K F R Q K Q Y L S I A E R A E F S S
Msx2 (Elc) M P F S V E A L A K N S W K E G Y S R S P C T L R K H K T N R K P R T P F T T S Q L L A L E R K F R Q K Q Y L S I A E R A E F S S
Msx2 (Amm) M P F S V E A L A A S S W K D G Y S R S P C T L R K H K T N R K P R T P F T T S Q L L A L E R K F R Q K Q Y L S I A E R A E F S N
MsxD (Fur) M P F S V E A L S R R S W T K S F S R S P C P L R K H K T N R K P R T P F T T S Q L L A L E R K F R Q K Q Y L S I A E R A E F S T
MsxD (Ten) M P F S V E A L S R S L W A R S F S R S P C P L R K H K T N R K P R T P F T T S Q L L A L E R K F R Q K Q Y L S I A E R A E F S T
MsxD (Dar) M P F S V E S L S K H P W M N S Y S R S P C P L R K H K T N R K P R T P F T T S Q L L A L E R K F R Q K Q Y L S I A E R A E F S S
MsxA (Dar) M P F S V D A L S D S A W N K P L S R S P C P L R K H K T N R K P R T P F T T A Q L L A L E R K F R Q K Q Y L S I A E R A E F S S
Msx3 (Mum) M P F S V E S L L G V P W P P V H S R S P C T L R K H K T N R K P R T P F T T A Q L L A L E R K F H Q K Q Y L S I A E R A E F S S
Msx3 (Ran) M P F S V E S L L G E P W P P V H S R S P C T L R K H K T N R K P R T P F T T A Q L L A L E R K F H Q K Q Y L S I A E R A E F S S
Msx3 (Mod) M P F S V E S L L G A P W P S A Y S R S P C T L R K H K N N R K P R T P F T T A Q L L A L E R K F R Q K Q Y L S I A E R A E F S S
MsxB (Dar) M P F S V E S L A D T E W N S S F T R S P C T L R K H K T N R K P R T P F S T S Q L L S L E R K F R Q K Q Y L S I A E R A E F S N
MsxB (Fur) M P F S V E S L S E T K W S H A Y T R S P C N L R K H K N N R K P R T P F T T S Q L L S L E R K F R Q K Q Y L S I A E R A E F S N
MsxC (Dar) M P F S V E S L S E S E W Q T T Y T R S P C T L R K H K N N R K P R T P F T T S Q L L A L E R K F R Q K Q Y L S I A E R A E F S N
MsxC (Fur) M P F S V E S L A E S P W Q T A F P R S P C N L R K H K N N R K P R T P F T T S Q L L A L E R K F R Q K Q Y L S I A E R A E F S N
MsxC (Ten) M P F S V E A L S E S P W Q T A F P R S P C N L R K H K N N R K P R T P F T T S Q L L A L E R K F R Q K Q Y L S I A E R A E F S N
Msx (Brf) M P F S V A S L A E G P W Q S S Y S R T P C T L R K H K T N R K P R T P F T T Q Q L L A L E R K F R Q K Q Y L S I A E R A E F S A
Msx (Sak) L T F S V A S L S E G P W D S S F S R S P C T L R K H K T N R K P R T P F T T S Q L L A L E R K F R Q K Q Y L S I A E R A E F S A
Msx (Nev) M S F S V E S L S E S S W H S T Y D R S P C Q L R K H K A N R K P R T P F T T S Q L L A L E R K F R Q K Q Y L S I A E R A E F S A
Msx (Acm) M S F S V E S L S E R P W H S T Y D R S P C Q L R K H K T N R K P R T P F T T S Q L L S L E R K F R Q K Q Y L S I A E R A E F S A

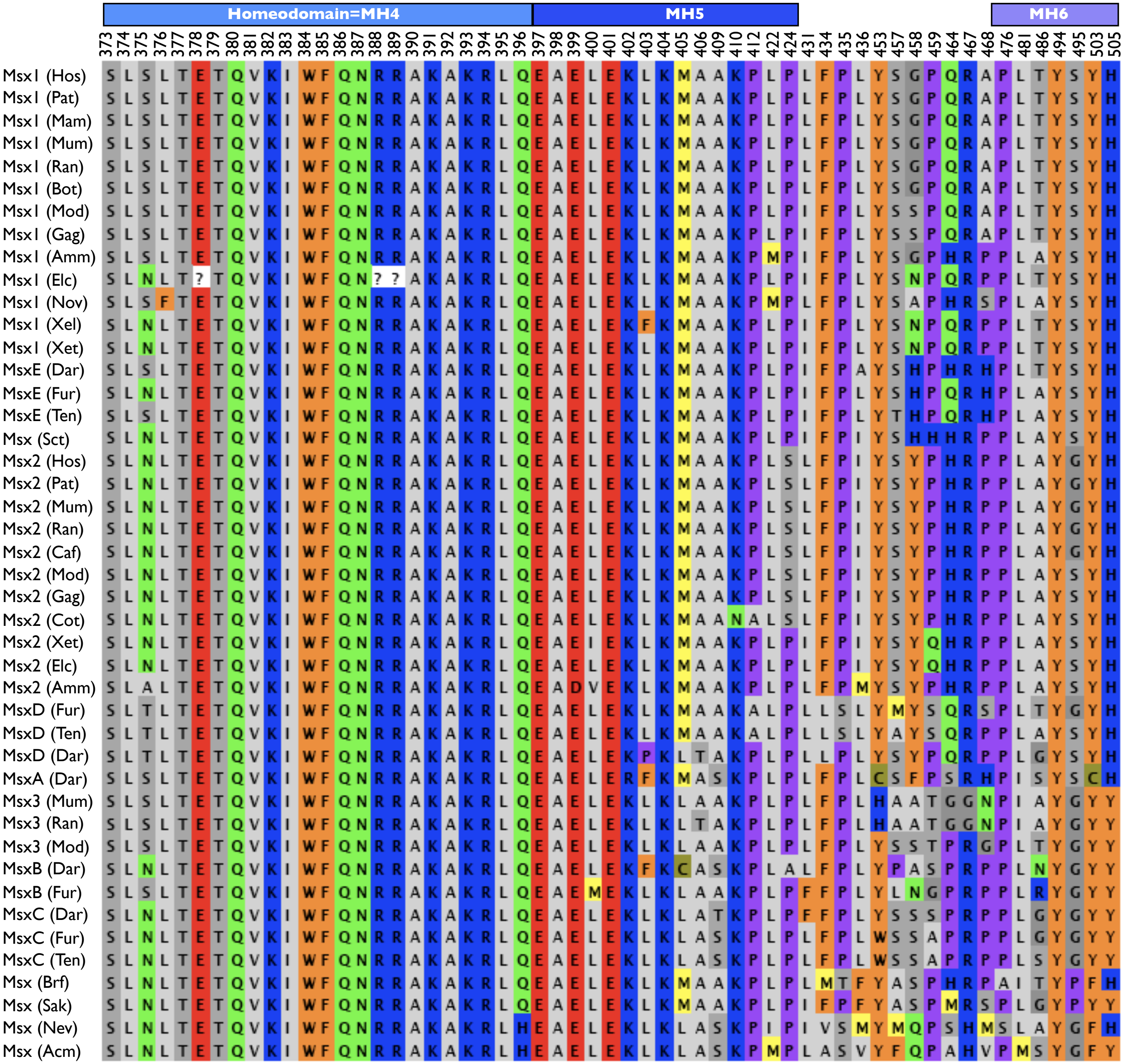
Homeodomain=MH4
MH5
MH6
373 374 375 376 377 378 379 380 381 382 383 384 385 386 387 388 389 390 391 392 393 394 395 396 397 398 399 400 401 402 403 404 405 406 409 410 412 422 424 431 434 435 436 453 457 458 459 464 467 468 476 481 486 494 495 503 505
Msx1 (Hos) SLSLTETQVKIWFQNRRAKAKRLQEAELEKLKMAAKPLPLFPLYSGPQRAPLTYSYH
Msx1 (Pat) SLSLTETQVKIWFQNRRAKAKRLQEAELEKLKMAAKPLPLFPLYSGPQRAPLTYSYH
Msx1 (Mam) SLSLTETQVKIWFQNRRAKAKRLQEAELEKLKMAAKPLPLFPLYSGPQRAPLTYSYH
Msx1 (Mum) SLSLTETQVKIWFQNRRAKAKRLQEAELEKLKMAAKPLPLFPLYSGPQRAPLTYSYH
Msx1 (Ran) SLSLTETQVKIWFQNRRAKAKRLQEAELEKLKMAAKPLPLFPLYSGPQRAPLTYSYH
Msx1 (Bot) SLSLTETQVKIWFQNRRAKAKRLQEAELEKLKMAAKPLPLFPLYSGPQRAPLTYSYH
Msx1 (Mod) SLSLTETQVKIWFQNRRAKAKRLQEAELEKLKMAAKPLPIFPLYSSPQRAPLTYSYH
Msx1 (Gag) SLSLTETQVKIWFQNRRAKAKRLQEAELEKLKMAAKPLPIFPLYSSPQRAPLTYSYH
Msx1 (Amm) SLSLTETQVKIWFQNRRAKAKRLQEAELEKLKMAAKPMPIFPLYSGPHRPPLAYSYH
Msx1 (Elc) SLNLT?TQVKIWFQN??AKAKRLQEAELEKLKMAAKPLPIFPLYSNPQRPPLTYSYH
Msx1 (Nov) SLSFTETQVKIWFQNRRAKAKRLQEAELEKLKMAAKPMPLFPLYSAPHRSPLAYSYH
Msx1 (Xel) SLNLTETQVKIWFQNRRAKAKRLQEAELEKFKMAAKPLPIFPLYSNPQRPPLTYSYH
Msx1 (Xet) SLNLTETQVKIWFQNRRAKAKRLQEAELEKLKMAAKPLPIFPLYSNPQRPPLTYSYH
MsxE (Dar) SLSLTETQVKIWFQNRRAKAKRLQEAELEKLKMAAKPLPIFPAYSHPHRHPLTYSYH
MsxE (Fur) SLNLTETQVKIWFQNRRAKAKRLQEAELEKLKMAAKPLPIFPLYSHPQRHPLAYSYH
MsxE (Ten) SLSLTETQVKIWFQNRRAKAKRLQEAELEKLKMAAKPLPIFPLYTHPQRHPLAYSYH
Msx (Sct) SLNLTETQVKIWFQNRRAKAKRLQEAELEKLKMAAKPLPIFPIYSHHHRPPLAYSYH
Msx2 (Hos) SLNLTETQVKIWFQNRRAKAKRLQEAELEKLKMAAKPLSLFPIYSYPHRPPLAYGYH
Msx2 (Pat) SLNLTETQVKIWFQNRRAKAKRLQEAELEKLKMAAKPLSLFPIYSYPHRPPLAYGYH
Msx2 (Mum) SLNLTETQVKIWFQNRRAKAKRLQEAELEKLKMAAKPLSLFPIYSYPHRPPLAYGYH
Msx2 (Ran) SLNLTETQVKIWFQNRRAKAKRLQEAELEKLKMAAKPLSLFPIYSYPHRPPLAYGYH
Msx2 (Caf) SLNLTETQVKIWFQNRRAKAKRLQEAELEKLKMAAKPLSLFPIYSYPHRPPLAYGYH
Msx2 (Mod) SLNLTETQVKIWFQNRRAKAKRLQEAELEKLKMAAKPLSLFPIYSYPHRPPLAYSYH
Msx2 (Gag) SLNLTETQVKIWFQNRRAKAKRLQEAELEKLKMAAKPLSLFPIYSYPHRPPLAYSYH
Msx2 (Cot) SLNLTETQVKIWFQNRRAKAKRLQEAELEKLKMAANALSLFPIYSYPHRPPLAYSYH
Msx2 (Xet) SLNLTETQVKIWFQNRRAKAKRLQEAEIEKLKMAAKPLPIFPIYSYQHRPPLAYSYH
Msx2 (Elc) SLNLTETQVKIWFQNRRAKAKRLQEAELEKLKMAAKPLPLFPIYSYQHRPPLAYSYH
Msx2 (Amm) SLALTETQVKIWFQNRRAKAKRLQEADVEKLKMAAKPLPLFPMYSYPHRPPLAYSYH
MsxD (Fur) SLTLTETQVKIWFQNRRAKAKRLQEAELEKLKMAAKALPLLSLYMYSQRSPLTYGYH
MsxD (Ten) SLTLTETQVKIWFQNRRAKAKRLQEAELEKLKMAAKALPLLSLYAYSQRPPLAYSYH
MsxD (Dar) SLTLTETQVKIWFQNRRAKAKRLQEAELEKPKLTAKPLPLLPLYSYPQRPPLGYSYH
MsxA (Dar) SLSLTETQVKIWFQNRRAKAKRLQEAELERFKMASKPLPLFPLCSFPSRHPISYSCH
Msx3 (Mum) SLSLTETQVKIWFQNRRAKAKRLQEAELEKLKLAAKPLPLFPLHAATGGNPIAYGYY
Msx3 (Ran) SLSLTETQVKIWFQNRRAKAKRLQEAELEKLKLTAKPLPLFPLHAATGGNPIAYGYY
Msx3 (Mod) SLSLTETQVKIWFQNRRAKAKRLQEAELEKLKLAAKPLPLFPLYSSTPRGPLTYGYY
MsxB (Dar) SLNLTETQVKIWFQNRRAKAKRLQEAELEKFKCASKPLALFPLYPASPRPPLNYGYY
MsxB (Fur) SLSLTETQVKIWFQNRRAKAKRLQEAEMEKLKLAAKPLPFFPLYLNGPRPPLRYGYY
MsxC (Dar) SLNLTETQVKIWFQNRRAKAKRLQEAELEKLKLATKPLPFFPLYSSSPRPPLGYGYY
MsxC (Fur) SLNLTETQVKIWFQNRRAKAKRLQEAELEKLKLASKPLPLFPLWSSAPRPPLGYGYY
MsxC (Ten) SLNLTETQVKIWFQNRRAKAKRLQEAELEKLKLASKPLPLFPLWSSAPRPPLSYGYY
Msx (Brf) SLNLTETQVKIWFQNRRAKAKRLQEAELEKLKMAAKPLPLMTFYASPHRPAITYPFH
Msx (Sak) SLNLTETQVKIWFQNRRAKAKRLQEAELEKLKMAAKPLPIFPFYASPMRSPLGYPYY
Msx (Nev) SLNLTETQVKIWFQNRRAKAKRLHEAELEKLKLASKPIPIVSMYMQPSHMSLAYGFH
Msx (Acm) SLNLTETQVKIWFQNRRAKAKRLHEAELEKLKLASKPMPLASVYFQPAHVPMSYGFY
