# Supplementary material for: Domain duplication, divergence, and loss events in vertebrate Msx paralogs reveal phylogenomically informed disease markers
Source: BMC Evol Biol. 2009 Jan 20;9:18. doi: 10.1186/1471-2148-9-18 (PMC2655272; doi:10.1186/1471-2148-9-18)

A) Nematostella Groucho1 gene exon structure within JGI_60 contig:

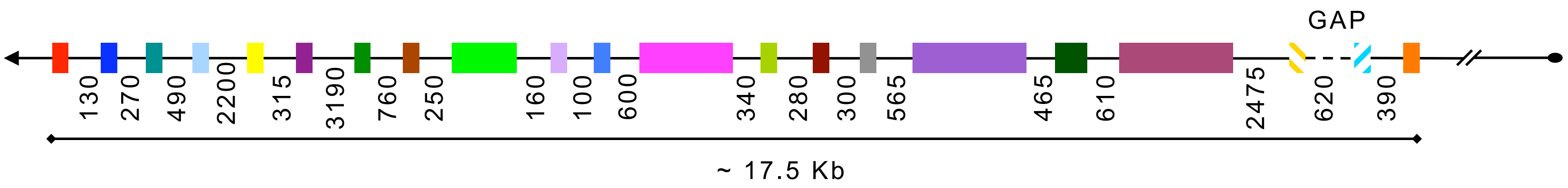


B) Nematostella Groucho1a gene exon structure within JGI_1316.

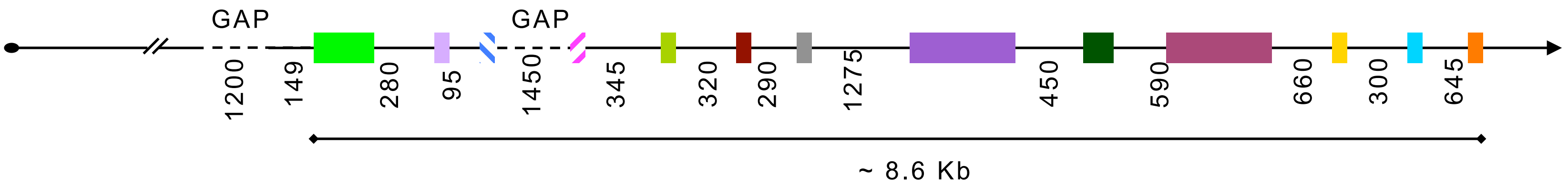


C) Known ESTs for Nematostella Groucho1 (top) and Groucho la (bottom).

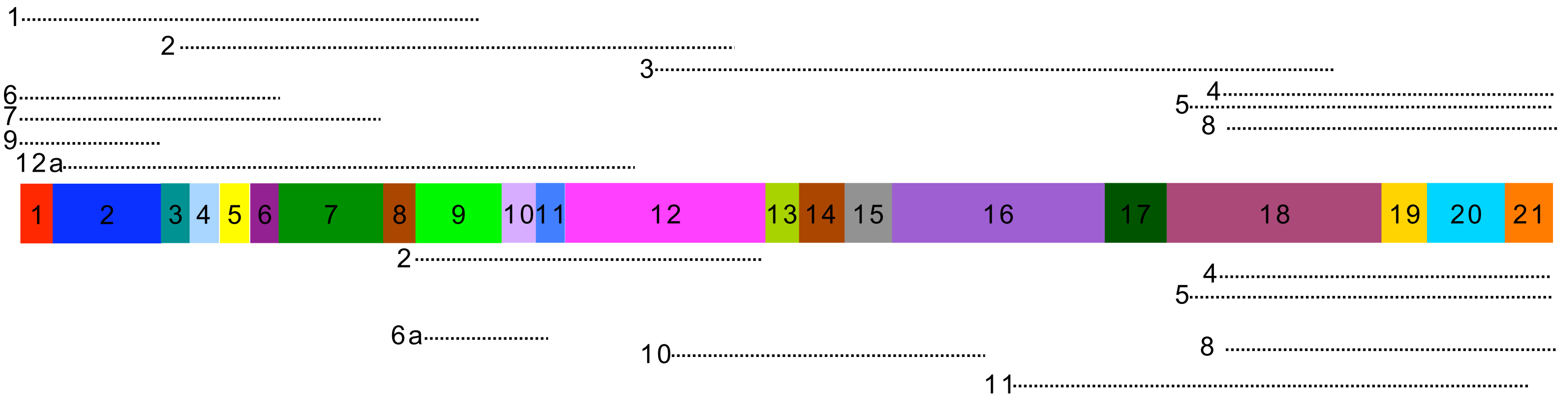

Supplement: Additional file 5 — Nematostella Groucho Loci with Exon Structure. This file illustrates the exon/intron map for the Nematostella Groucho1 and Groucho1a genes and their correspondence to known Nematostella ESTs. [file 1471-2148-9-18-S5.pdf]
