# Supplementary material for: Domain duplication, divergence, and loss events in vertebrate Msx paralogs reveal phylogenomically informed disease markers
Source: BMC Evol Biol. 2009 Jan 20;9:18. doi: 10.1186/1471-2148-9-18 (PMC2655272; doi:10.1186/1471-2148-9-18)
Supplement: Additional file 6 — Pairwise Evolutionary Distance Calculations for MSX1 and MSX2 compared to Shark Msx1, Lamprey MsxA and Amphioxus Msx. This file displays the pairwise evolutionary distance calculations of Msx domain subsets against different outgroup sequences. [file 1471-2148-9-18-S6.doc]

**Additional File 6. Pairwise evolutionary distance results for MSX1 and MSX2 compared to Shark Msx1, Lamprey MsxA and Amphioxus Msx outgroup sequences.**

Row and column lables below: [1] #HsMSX1

[2] #HsMSX2

[3] #StMsx

[4] #PmMsxA

[5] #BfMsx

**A) Complete coding sequence alignment - Pairwise Distance Matrix:**

[ 1 2 3 4 5 ]

**All domains and intervals**; Rates among sites=different, gamma=1.0; #sites=224

[1]

[2] 0.41 [0.06]

[3] 0.31 [0.05] 0.42 [0.06]

[4] 0.51 [0.07] 0.54 [0.08] 0.60 [0.08]

[5] 0.47 [0.06] 0.60 [0.08] 0.65 [0.09] 0.70 [0.09]

**B) ED mutation domains - Pairwise Distance Matrices:**

[ 1 2 3 4 5 ]

**Nterm**; Rates among sites=different, gamma=1.0; # sites=26

[1]

[2] 1.76 [0.77]

[3] 2.61 [1.54] 2.05 [0.90]

[4] 1.86 [0.89] 1.39 [0.56] 2.75 [1.41]

[5] 0.86 [0.35] 1.55 [0.63] 2.35 [1.37] 1.84 [0.88]

**MH1N, MH4**; uniform rates; # sites=73

[1]

[2] 0.04 [0.02]

[3] 0.04 [0.02] 0.00 [0.00]

[4] 0.10 [0.03] 0.05 [0.03] 0.05 [0.03]

[5] 0.07 [0.03] 0.07 [0.03] 0.07 [0.03] 0.09 [0.03]

**C) OFC mutation domains - Pairwise Distance Matrices:**

[ 1 2 3 4 5 ]

**iMH1C, MH2, MH3**; Rates among sites=different, gamma=1.0; #sites=69

[1]

[2] 0.68 [0.16]

[3] 0.39 [0.11] 0.86 [0.19]

[4] 0.96 [0.24] 1.28 [0.31] 1.16 [0.28]

[5] 0.87 [0.20] 1.26 [0.30] 1.39 [0.32] 1.51 [0.33]

**iMH5-6, MH6**; Rates among sites=different, gamma=1.0; #sites=39

[1]

[2] 0.61 [0.19]

[3] 0.33 [0.13] 0.52 [0.15]

[4] 0.64 [0.20] 0.70 [0.20] 0.92 [0.25]

[5] 1.05 [0.29] 1.16 [0.36] 1.10 [0.32] 1.23 [0.34]

i**MH1C, MH2, MH3, iMH5-6, MH6**; Rates among sites=different, gamma=1.0; #sites=15

[1]

[2] 0.65 [0.12]

[3] 0.37 [0.08] 0.73 [0.12]

[4] 0.83 [0.15] 1.04 [0.18] 1.07 [0.19]

[5] 0.93 [0.17] 1.22 [0.23] 1.28 [0.23] 1.40 [0.24]
