# Supplementary material for: Domain duplication, divergence, and loss events in vertebrate Msx paralogs reveal phylogenomically informed disease markers
Source: BMC Evol Biol. 2009 Jan 20;9:18. doi: 10.1186/1471-2148-9-18 (PMC2655272; doi:10.1186/1471-2148-9-18)
Supplement: Additional file 7 — Msx Domain definition alignments for Pairwise Distance Calculations. This file displays the domain definitions used in the Pairwise Distance Calculations. [file 1471-2148-9-18-S7.doc]

**Additional File 7. Msx domain definition alignments for pairwise distance calculations.**

#MEGA

!Format

DataType=Protein

NSeqs=5 NSites=392

Identical=. Missing=? Indel=-;

!Domain=Nterm;

[ 1 1111111112 2222222223 3333333334 4444444445 5555555556 6666666667 7777777778 8888888889 ]

[ 1234567890 1234567890 1234567890 1234567890 1234567890 1234567890 1234567890 1234567890 1234567890 ]

#HsMSX1 M-T---S--L PLGVKVEDSA ------FG-- --K-PAGGGA GQ-A------ ------P--S AAAA------ --------T- --AAAM-GAD

#HsMSX2 M-A---S--- P--SK-GNDL ------FSPD -EEGPA---V VA-G------ ------P--G PG-------- --------P- --GGAE-GAA

#StMsx M--------L Q--LE-RDRL ------YM-- --A-PCS--V MS-A------ ------PQVT TGGVRS---- ------DEPR SLANVKLAAA

#PmMsxA MNAFRFAVEE D--AK-SHKL SPTPDSFASD AEQSPA---A MQDGKAAREE LSSSHSP--S SSSSSSTTSS SSASLSSSP- --SGLD-VAA

#BfMsx M-AQ--ST-L P--TS-SNSA ------FS-- --K-PT---T -S-A------ ------P--S SASS------ --------P- --TST-----

[ 1 11]

[ 9999999990 00]

[ 1234567890 12]

#HsMSX1 EEGAKPKVSP SL

#HsMSX2 EE-RRVKVSS --

#StMsx EEMDRPK-AP --

#PmMsxA KS-AR-SVCA --

#BfMsx E---K----- --

!Domain=MH1N;

[ 1111111111 11111]

[ 0000000111 11111]

[ 3456789012 34567]

#HsMSX1 LPFSVEALMA DHRKP

#HsMSX2 LPFSVEALMS D-KKP

#StMsx LPFSVEALMS D-RKP

#PmMsxA LPFSVESLMS D-RRP

#BfMsx LPFSVASLMA D--KP

!Domain=iMH1C;

[ 1111111111 1111111111 1111111111 1111111111 1111111111 1111111111 1111111111 1111111111 1122222222 ]

[ 1122222222 2233333333 3344444444 4455555555 5566666666 6677777777 7788888888 8899999999 9900000000 ]

[ 8901234567 8901234567 8901234567 8901234567 8901234567 8901234567 8901234567 8901234567 8901234567 ]

#HsMSX1 GAKE-SALAP SEGVQAA-GG SA-QPLGVP- -PG--SLGAP D--APSSPR- PLGH---FSV GGLL-KLP-- ---EDALV-K AESP--E--K

#HsMSX2 -PKEASPL-P AES--AS-AG ATLRPLLLS- --G---HGAR ---EAHSPG- P--------- --LV-K-PF- ---ETASV-K SENS--E---

#StMsx -SRE-RAA-- SE---AALGG TS-QSLS-PR MAGQETAATP ---LAATT-- S------YTV EGLL-KIS-- ---EEALV-K SESG--E--R

#PmMsxA -SRD-SAL-- RS---AS-DG TA-GDRS-P- --G------P ---LPASS-- P------YSV KSLLHRGPTG PGPQSHCA-D GGEPCAEG-D

#BfMsx --KE-TEQ-- NQ---SD-SG PP--PLQSP- -GGPQSPASP PATVPTAPAQ PPSRPSDFSV EGILSK-PCS S--ETAAAEK GHDP--TGFA

[ 2222]

[ 0011]

[ 8901]

#HsMSX1 P-ER

#HsMSX2 --DG

#StMsx Q-ER

#PmMsxA PQDG

#BfMsx A-AR

!Domain=MH2;

[ 2222222222 22]

[ 1111111122 22]

[ 2345678901 23]

#HsMSX1 TP-WMQSP-R FS

#HsMSX2 AA-WMQEPGR YS

#StMsx TP-WMQDP-R FS

#PmMsxA LAGWMHGAA- YS

#BfMsx FP-WLQSS-R YS

!Domain=MH3;

[ 2222222222 2222222222 2]

[ 2222223333 3333334444 4]

[ 4567890123 4567890123 4]

#HsMSX1 PPPAR-RL-S PPACTLRKHK T

#HsMSX2 PPP-R-HM-S PTTCTLRKHK T

#StMsx PPP-R-RM-S PPACTLRKHK T

#PmMsxA PPP-R-RL-S PPACTLRKHK A

#BfMsx PPP-RDRLPT PNKCTLRKHK T

!Domain=MH4;

[ 2222222222 2222222222 2222222222 2222222222 2222222222 2222233333 ]

[ 4444455555 5555566666 6666677777 7777788888 8888899999 9999900000 ]

[ 5678901234 5678901234 5678901234 5678901234 5678901234 5678901234 ]

#HsMSX1 NRKPRTPFTT AQLLALERKF RQKQYLSIAE RAEFSSSLSL TETQVKIWFQ NRRAKAKRLQ

#HsMSX2 NRKPRTPFTT SQLLALERKF RQKQYLSIAE RAEFSSSLNL TETQVKIWFQ NRRAKAKRLQ

#StMsx NRKPRTPFTT SQLLALERKF RQKQYLSIAE RAEFSSSLNL TETQVKIWFQ NRRAKAKRLQ

#PmMsxA SRKPRTPFTT SQLLALERKF RQKQYLSIAE RAEFSNSLNL TETQVKIWFQ NRRAKAKRLQ

#BfMsx NRKPRTPFTT QQLLALERKF RQKQYLSIAE RAEFSASLNL TETQVKIWFQ NRRAKAKRLQ

!Domain=MH5;

[ 3333333333 3333333333 ]

[ 0000011111 1111122222 ]

[ 5678901234 5678901234 ]

#HsMSX1 EAELEKLKMA A--KP-MLPP

#HsMSX2 EAELEKLKMA A--KP-MLPS

#StMsx EAELEKLKMA A--KP-MLPP

#PmMsxA EAELEKLKMA AAAKP-LLTP

#BfMsx EAELEKLKMA A--KP-MLPP

!Domain=iMH5-6;

[ 3333333333 3333333333 3333333333 33333]

[ 2222233333 3333344444 4444455555 55555]

[ 5678901234 5678901234 5678901234 56789]

#HsMSX1 AAFGLS--F- PLGGPAAVAA AAGASLYG-A S-GP-

#HsMSX2 -SFSLP--F- PISSP--LQA A---SIYG-A S-YP-

#StMsx -AFGIS--F- PIGTP--VPA T---SLYG-A S-HH-

#PmMsxA AGFGLPFQF- --GAP----- -----LYATA SVSPA

#BfMsx -ALGMT--F- P--SP----- -----FYA-A A-SP-

!Domain=MH6;

[ 3333333333 3333333333 3333333333 333]

[ 6666666666 7777777777 8888888888 999]

[ 0123456789 0123456789 0123456789 012]

#HsMSX1 FQRAA-LPV- A-PVGLYT-- -AHVGY-SMY HLT

#HsMSX2 FHRPV-LPI- P-PVGLYA-- -TPVGY-GMY HLS

#StMsx FHRPT-LPV- S-PVGLYA-- -AHVGY-SMY HLA

#PmMsxA FPRAA-LPMG --HLGLYS-- -APMGY-GMY PLS

#BfMsx FHRPG-LPVQ ACQIGPYTYY PSHT-YAGFI HSS
