# Supplementary material for: Domain duplication, divergence, and loss events in vertebrate Msx paralogs reveal phylogenomically informed disease markers
Source: BMC Evol Biol. 2009 Jan 20;9:18. doi: 10.1186/1471-2148-9-18 (PMC2655272; doi:10.1186/1471-2148-9-18)
Supplement: Additional file 8 — Table of MAPP scores for Msx1 alignments inclusive of Human to Cnidarians, Tetrapods or Amniotes. This file displays the Multivariate Analysis of Protein Polymorphism (MAPP) Scores of different known human MSX1 missense coding variants within different phylogenetic depths. [file 1471-2148-9-18-S8.doc]

Additional File 8. Table of MAPP scores for Msx1 alignments inclusive of Human to Cnidarians, Tetrapods or Amniotes.

|  | MAPP Values | | |
| --- | --- | --- | --- |
| MSX1 Coding Variants | Cnidarian – Human | Tetrapod – Human | Amniote – Human |
| G16D | 25.02 | 24.62 | 39.53 |
| A23V | N/A | N/A | 2.95 |
| A34G | N/A | N/A | 22.29 |
| M61K | 35.76 | 27.72 | 22.72 |
| E78V | 4.54 | 5.15 | 25.4 |
| G91D | N/A | N/A | 35.86 |
| G98E | N/A | 37.88 | 40.11 |
| V114G | 12.69 | 9.89 | 23.22 |
| G116E | 4.69 | 39.92 | 40.07 |
| P147Q | 10.36 | 17.67 | 17.67 |
| R151S | 6.16 | 21.57 | 21.57 |
| R196P | 40.70 | 40.70 | 40.70 |
| A219T | 38.15 | 33.24 | 33.24 |
| A221E | 37.64 | 37.64 | 37.64 |
| G267C | 8.82 | 16.54 | 15.25 |
| P278S | 5.79 | 17.5 | 17.67 |
